# ADAR1-circRAB5A-BIP axis governs radiotherapy resistance in colorectal cancer through coordinating protective autophagy and apoptosis

**DOI:** 10.1080/15384047.2026.2677975

**Published:** 2026-06-21

**Authors:** Wangsheng Chen, Xu Zhang, Zhengfei Zhao, Wang Xin, Jianxin Li, Xiangming Che

**Affiliations:** a Department of General Surgery, the First Affiliated Hospital of Xi'an Jiaotong University, Xi'an, Shanxi, China; b Department of General Surgery (Gastrointestinal Surgery), the Affiliated Hospital of Southwest Medical University, Sichuan, China; c Department of Geriatrics, the Affiliated Hospital of Southwest Medical University, Sichuan, China

**Keywords:** Colorectal cancer, radioresistance, circular RNA, autophagy, ubiquitination, apoptosis

## Abstract

**Background:**

Colorectal cancer (CRC) ranks among the most prevalent malignancies globally, and radiotherapy remains a critical treatment modality. However, its efficacy is frequently compromised by acquired radioresistance. The endoplasmic reticulum chaperone protein BIP plays a pivotal role in regulating radioresistance by coordinating the balance between protective autophagy and apoptosis, though the regulatory roles of circular RNAs (circRNAs) in this process remain poorly understood.

**Materials:**

Differentially expressed circRAB5A (hsa-circ-0123297) was identified from the GSE186940 dataset. Its expression was validated in radioresistant CRC clinical samples and cell lines. Mechanistic investigations involved ADAR1 binding assays, circRAB5A gain/loss-of-function studies, autophagy-apoptosis profiling, ubiquitination analysis, TRIM21-mediated degradation assays, and *in vivo* xenograft models.

**Results & conclusion:**

CircRAB5A was significantly downregulated in radioresistant CRC clinical samples and cell lines. This downregulation was driven by ADAR1, which suppressed circRAB5A biogenesis by binding to Alu Jo/Jr elements. Functional assays showed circRAB5A depletion conferred radioresistance in CRC cells by promoting protective autophagy and inhibiting apoptosis. Mechanistically, circRAB5A destabilized BIP by enhancing TRIM21-mediated ubiquitination. The circRAB5A/BIP axis further modulates the autophagy-apoptosis balance through the p-Akt/Beclin1 signaling pathway, thereby influencing radiosensitivity. *In vivo* xenograft experiments demonstrated that stable knockdown of circRAB5A attenuated the anti-tumor effects of radiation, whereas knockdown of BIP sensitized CRC cells to radiotherapy even at low doses. Collectively, the ADAR1/circRAB5A/BIP molecular circuitry governs CRC radioresistance by regulating the autophagy-apoptosis balance. Our findings highlight that low circRAB5A expression may serve as a potential biomarker for radioresistance, and that targeting this axis, particularly BIP, represents a promising strategy for overcoming radioresistance in CRC.

## Introduction

Colorectal cancer (CRC) is the third most common malignancy globally, with more than 1.5 million new cases annually.^1^ It plays a crucial role in locoregional disease management and has attracted significant attention in recent years.^2^ ^,^ ^3^ Despite technological advancements, intrinsic or acquired radioresistance affects 30%–40% of patients,^4^ leading to recurrence and poor survival. While dysregulation of DNA repair pathways and oncogenic signaling has been implicated, emerging evidence highlights non-coding RNAs,^5^ ^,^ ^6^ particularly circular RNAs (circRNAs), as pivotal yet underexplored modulators of radiation responses.^7^ CircRNAs, formed by back-splicing of pre-mRNA, exhibit tissue-specific expression and regulate cancer progression by sponging microRNAs (miRNAs), interacting with RNA-binding proteins, or even encoding peptides.^8^ ^,^ ^9^ Dysregulation of certain circRNAs always modulates signaling pathways by affecting the function of its target miRNA or proteins, thereby exhibiting the function of regulatory RNAs.^10^ Accumulating evidence has revealed their roles in chemoresistance and immune evasion,^11^ their involvement in radioresistance remains largely uncharacterized.

Endoplasmic reticulum (ER) stress adaptation has emerged as a survival mechanism in radioresistant tumors.^12^ The chaperone BIP, a master regulator of the unfolded protein response (UPR), stabilizes proteostasis, which modulates both pro-survival autophagy and apoptosis under extracellular stress.^13^ The role of BIP in balancing autophagy and apoptosis is a double-edged sword,^14^ which in some situations, BIP enhances autophagy and recycling of the UPR to maintain cell survival and inhibit apoptosis, which contributes to radioresistance.^15^ However, over-activated autophagy also leads to uncontrolled apoptosis. Therefore, revealing the role of autophagy in radioresistance is key to uncovering the mechanisms of BIP-mediated autophagy-apoptosis balance. Specifically, in CRC, the function of BIP, its relationship with autophagy, and the involvement of regulatory circRNAs in radioresistance warrant further exploration.

Adenosine deaminase acts on RNA 1 (ADAR1), an RNA-editing enzyme that catalyzes adenosine-to-inosine (A-to-I) conversions.^16^ As reported, overexpression of ADAR1 in tumors promotes metastasis and therapy resistance by altering mRNA splicing or microRNA (miRNA) targeting.^17^ Notably, Alu elements, which are primate-specific repetitive sequences that facilitate circRNA biogenesis, are hotspots for ADAR1 editing. ADAR1 can regulate the biogenesis of circRNAs by modulating Alu sequences, thereby promoting differential expression.^18^ A study by Tian et al. also highlighted that ADAR1 was upregulated during radioresistance^19^; however, its underlying molecular mechanism and its regulation of circRNAs are still unclear.

Herein, we address these gaps by investigating the role of a novel circRNA, circRAB5A, and its regulatory network involving ADAR1 and BIP in CRC radioresistance. Using the Gene Expression Omnibus (GEO) dataset and bioinformatics analysis, we hypothesized that ADAR1 suppresses circRAB5A biogenesis by binding to Alu Jo/Jr elements in radioresistant CRC cells. The deficiency circRAB5A modulates BIP stability through TRIM21-mediated ubiquitination, thereby coordinating the autophagy-apoptosis balance to drive radioresistance. Our findings provide new insights into the molecular basis of the ADAR1/circRAB5A/BIP axis in CRC radioresistance and highlight potential therapeutic targets to enhance the efficacy of radiotherapy.

## Materials and methods

### Patient samples

Forty pairs of CRC tumor tissues and adjacent normal colorectal mucosal tissues were collected from patients who underwent radical resection at the Department of Gastrointestinal Surgery, Southwest Medical University (Luzhou, Sichuan, China), between September 2022 and September 2023. All patients received preoperative radiotherapy (45–50 Gy in 25–28 fractions) as part of their treatment regimen, and none of them had received neoadjuvant chemotherapy or other anti-cancer therapies prior to surgery. The radiosensitive and radioresistant groups were classified as follows. Patients were stratified into radiosensitive (*n* = 20) and radioresistant (*n* = 20) groups based on clinical outcomes. Radioresistance was defined as local tumor recurrence within six months post-radiotherapy, confirmed by endoscopic or imaging (CT or MRI) evidence. Radiosensitive patients showed no recurrence and achieved a complete or partial response as assessed by the RECIST 1.1 criteria^20^ within 12 months post-radiotherapy. The study was approved by the Ethics Committee of Southwest Medical University (Approval No. KY2022025) and was conducted in accordance with the Declaration of Helsinki. Written informed consent was obtained from all the participants.

### Cell culture and reagents

The human colorectal cancer cell lines SW480 (ATCC CCL-228), SW620 (ATCC CCL-227), HCT8 (ATCC CCL-244), HCT116 (ATCC CCL-247), and Caco-2 (ATCC HTB-37), and the normal human colon epithelial cell line NCM460 (CELLCOOK, CCC-223) were purchased from Guangzhou CELLCOOK Biotech Co. (Guangzhou, China). The cell lines were authenticated by short tandem repeat profiling (Genetic Testing Biotechnology Co., Guangzhou, China). The cells were maintained in RPMI 1640 medium (Gibco, USA) supplemented with 10% fetal bovine serum (FBS, Gibco), 100 U/mL penicillin, and 100 μg/mL streptomycin (Gibco) at 37 °C in a humidified incubator with 5% CO₂. All the cells tested negative for mycoplasma contamination prior to harvest.

For cell autophagy analysis, the autophagy inhibitor, chloroquine (CQ) (CAS No. 54-05-7) was purchased from Selleck (USA) and used at a working concentration of 10 μM. The autophagy inducer, Rapamycin (Rapa) (CAS No. 53123-88-9) was purchased from Selleck and used at a work concentration of 25 nM. MG132 (Sigma-Aldrich) was used as a proteasome inhibitor at a concentration of 10 μM. The cultured cells were treated for 6 h to block proteasomal degradation.

### Construction of radiation-resistant cells

Irradiation-resistant SW480 cells (SW480-IR) were established using a fractionated irradiation protocol to mimic clinical radiotherapy regimens. Parental SW480 cells were cultured to 60% confluency in 10-cm dishes (Corning, USA) and exposed to 2 Gy per fraction at a dose rate of 0.75 Gy/min. After each irradiation, the cells were returned to the incubator (37 °C, 5% CO₂) and allowed to recover to 80% confluency before the next fraction. This process was repeated for 10 fractions, delivering a total cumulative dose of 20 Gy over five weeks (two fractions per week). After the final irradiation, the cells were cultured for an additional 3 weeks to stabilize the radioresistant phenotype.

### Clonogenic survival assay

Radiosensitivity was assessed using clonogenic survival assay. Briefly, SW480 and SW480-IR cells were seeded at a low density of 1000 cells per well in a 6-well plate and exposed to 0, 2, 4, 6, or 8 Gy radiation. After 14 d, colonies (>50 cells) were fixed with 4% paraformaldehyde (PFA), stained with 0.5% crystal violet, and counted using the ImageJ software. Dose‒response curves were generated using the multi-target single-hit model (y = (1 − *e* ^−^^*D*/*D*0^)^*n*^) in GraphPad Prism 9.0, where *D* represents the radiation dose (Gy), D0 represents the mean lethal dose (Gy), and *n* represents the extrapolation number. All experiments were performed with at least three independent biological replicates.

### Plasmid construction and cell transfection

The full-length linear sequence of circRAB5A was PCR-amplified from SW480 cDNA. The amplicon, supplemented with circular sequences, was cloned into the EcoRI/BamHI sites of the pLCDH-mir vector (GeneChem, Shanghai, China) using T4 DNA ligase (NEB). The recombinant plasmid (pLCDH-circRAB5A) was verified using Sanger sequencing (Tsingke Biotechnology Co., Beijing, China). The construction of ADAR1, BIP, and TRIM21 expression vectors, as well as BIP wild-type or truncated recombinant expression vectors, were conducted by Tsingke Biotechnology Co. by cloning the full-length or truncated sequence into the pcDNA3.1 vector (Addgene). The mock vector served as a negative control (NC).

For knocking down, small interfering RNAs (siRNAs) targeting circRAB5A (sicircRAB5A), ADAR1 (siADAR1), BIP (siBIP), and TRIM21 (siTRIM21) were designed and synthesized by GeneChem. Off-target effects were minimized by selecting sequences with fewer than two mismatches with other human transcripts. The siRNA sequences are provided in Supplementary Table S1.

For plasmid transfection, SW480 and SW620 cells were seeded in 6-well plates (Corning) at a density of 3 × 10⁵ cells/well and cultured to 60%–70% confluency. Transfection was performed using Lipofectamine 3000 (Invitrogen) according to the manufacturer's instructions. For siRNA transfection, cells were seeded at 2 × 10⁵ cells/well in 6-well plates and transfected with 50 nM siRNA using Lipofectamine RNAiMAX (Invitrogen) according to the manufacturer's instructions. The cells were harvested 48 h post-transfection for downstream assays.

### Identification of circRNAs

To characterize the circular structure of circRAB5A, multiple assays were conducted, including divergent/convergent PCR, RNase R digestion, oligo (dT) reverse transcription assay, and actinomycin D (ACT-D) assay, according to the published literature by Zhou et al.^21^

### Subcellular fractionation

Cytoplasmic and nuclear fractions were isolated using NE-PER Nuclear and Cytoplasmic Extraction Reagents (Thermo Scientific) following the manufacturer's protocol. qRT-PCR was performed to detect circRAB5A expression, using cytoplasmic (GAPDH) and nuclear (U6) markers used as controls.

### Cell apoptosis assay

Apoptosis was assessed using an Annexin V-FITC/PI Apoptosis Detection Kit (Solarbio, Beijing, China). The cells (3 × 10⁵/well in 6-well plates) were irradiated and harvested 24 h later. The cells were collected, washed, and resuspended in binding buffer. After being stained with 2.5 μL Annexin V-FITC and 2.5 μL PI, apoptotic cells were analyzed by flow cytometry (NovoCyte, Agilent, USA).

### Western blot (WB)

Total protein was extracted from SW480 and SW620 cells using RIPA lysis buffer (Beyotime, Jiangsu, China) supplemented with a protease inhibitor cocktail (Beyotime) and phosphatase inhibitor cocktail (Beyotime). The protein concentration was quantified using the BCA protein assay kit (Beyotime). Equal amounts of proteins were loaded and separated by 7.5%–15% SDS–PAGE gel and then transferred to PVDF membranes (Millipore, USA). The membranes were blocked with 5% non-fat milk or 3% FBS for 1 h at room temperature. Primary antibodies were incubated overnight at 4 °C: anti-BIP (Proteintech, 1:1000), anti-LC3B (CST, 1:2000), anti-p62 (CST, 1:2000), anti-p-Akt (CST, 1:1000), anti-Beclin1 (CST, 1:1000), anti-Flag (Sigma-Aldrich, 1:5000) and anti-GAPDH (Abclonal, 1:5000). After that, the membranes were washed with TBST and incubated with HRP-conjugated secondary antibodies (Abclonal, 1:5000) for 2 h at room temperature. Finally, the protein bands were visualized using ECL Plus reagent (Bio-Rad, USA) and imaged using a ChemiDoc Imaging System (Bio-Rad). Quantification was performed using the ImageJ software, with the target protein levels normalized to those of GAPDH.

### qRT-PCR assay

Total RNA was extracted from SW480 and SW620 cells using TRIzol reagent (Invitrogen) according to the manufacturer's protocol. Briefly, 1 × 10⁶ cells were lysed in 1 mL of TRIzol, incubated for 5 min at room temperature, and mixed with 200 μL of chloroform. After centrifugation (12,000 × *g* for 15 min), the aqueous phase was collected, and the RNA was precipitated with isopropanol, washed with 75% ethanol, and resuspended in RNase-free water. The RNA purity (A260/A280 ≥ 1.8) and concentration were measured using a NanoDrop 2000 spectrophotometer (Thermo Fisher Scientific). Next, 1 μg of RNA was reverse-transcribed into cDNA using the PrimeScript RT Reagent Kit with Genomic DNA (gDNA) Eraser (Takara). The reaction mixture included 2 μL 5× gDNA Eraser Buffer, 1 μL gDNA Eraser, and the RNA template and was incubated at 42 °C for 2 min to remove genomic DNA. Reverse transcription was performed with 1 μL of PrimeScript RT Enzyme Mix, 1 μL RT Primer Mix, and 4 μL 5 × RT Buffer, and incubated at 37 °C for 15 min and 85 °C for 5 s. qPCR was performed using TB Green Premix Ex Taq II (Takara) on a Bio-Rad CFX96 Real-Time System. Each reaction contained 10 μL TB Green Mix, 0.8 μL 10 μM forward/reverse primers, 2 μL cDNA, and 7.2 μL RNase-free water. The cycling conditions was set as follow: 95 °C for 30 s, followed by 40 cycles of 95 °C for 5 s and 60 °C for 30 s. Relative expression was calculated using the 2^⁻ΔΔCt^ method and normalized to that of GAPDH. The primer sequences for specific genes and internal controls are listed in Supplementary Table S2.

### Fluorescence imaging for autophagy detection

Autophagosome formation was visualized using mCherry-GFP-LC3 dual-fluorescence reporter (Beyotime). SW480 cells were transfected with the pCMV-mCherry-GFP-LC3 plasmid using Lipofectamine 3000 (Invitrogen) 24 h prior to radiation treatment (4 Gy). The cells were fixed with 4% paraformaldehyde 24 h post-irradiation and imaged using a Zeiss fluorescence microscope (Leica Microsystems) with a 63 oil immersion objective. Autolysosomes (red puncta, mCherry+GFP−) and autophagosomes per cell (yellow puncta, mCherry+GFP+) were counted manually from three random fields using the ImageJ software.

### Fluorescence imaging and fluorescence in situ hybridization (FISH) of circRAB5A

CircRAB5A subcellular localization was detected using a FISH kit (Riobio, Guangzhou, China)^22^ with a Cy5-labeled DNA probe targeting the circRAB5A back-splice junction. The cells were seeded on cover-slips, fixed with 4% PFA, permeabilized with 0.5% Triton X-100 (Beyotime), and hybridized with a 50 nM probe in hybridization buffer at 37 °C overnight. After washing with 2 × SSC, the nuclei were stained with DAPI. Images were acquired using a fluorescence microscope (Zeiss).

### Bioinformatics analysis

The prediction of RNA-binding proteins with Alu Jo/Jr sequences were performed using catRAPID online bioinformatics software^23^ according to the manufacturer's instructions. The prediction results were provided in Supplementary Tables S3 and S4.

### RNA pull-down

RNA-protein interactions were detected using the Pierce Magnetic RNA-Protein Pull-Down Kit (Thermo Scientific) with biotinylated probes targeting circRAB5A (GenePharma, Guangzhou, China). In brief, 1 × 10⁷ SW480 or SW620 cells were lysed in 1 mL of RNA lysis buffer with protease inhibitor cocktail and 100 U/mL RNase inhibitor (Takara) for 15 min on ice. Biotinylated probes were then mixed with 500 μL of cell lysate and incubated at 37 °C for 1 h to allow RNA-protein binding. Next, streptavidin magnetic beads were added to the probe-lysate mixture. The samples were rotated at 4 °C for 2 h to capture the biotinylated complexes. The beads were then washed with wash buffer to remove non-specific binding. The bound proteins were eluted with 50 μL of elution buffer at 95 °C for 5 min and analyzed by WB. NC probe pull-down served as a negative control.

### RNA immunoprecipitation (RIP) assay

RNA-protein interactions were validated using the Magna RIP RNA-Binding Protein Immunoprecipitation Kit (Millipore) following the manufacturer's protocol. In brief, 1 × 10⁷ SW480 or SW620 cells were lysed in 1 mL RIP lysis buffer containing protease inhibitor cocktail and 100 U/mL RNase inhibitor for 30 min on ice. The magnetic beads were then washed with RIP wash buffer and incubated with 5 μg anti-BIP antibody (CST) or normal IgG (Millipore, negative control) at 4 °C for 2 h. Next, the lysates (500 μL) were added to the antibody-bound beads and rotated at 4 °C overnight to co-immunoprecipitate the RNA‒protein complexes. The next day, the beads were washed with RIP wash buffer, and bound RNA was extracted using TRIzol reagent (Invitrogen) and analyzed by qRT-PCR.

### Ubiquitination assay

BIP ubiquitination was assessed using co-immunoprecipitation (co-IP) to detect ubiquitin conjugation. Briefly, SW480 cells were transfected with a circRAB5A overexpression plasmid, sicircRAB5A, or respective controls for 48 h. To block proteasomal degradation, the cells were treated with 10 μM MG132 (Sigma-Aldrich) for 6 h prior to lysis. The cells were then lysed in 500 μL IP lysis buffer (Beyotime) supplemented with a protease inhibitor cocktail, phosphatase inhibitor cocktail, and 10 mM N-ethylmaleimide (Sigma‒Aldrich). The lysates were incubated on ice for 30 min and then centrifuged at 14,000 × *g* for 15 min at 4 °C, after which the supernatants were collected. Next, 500 μg of total protein was pre-cleared with 20 μL protein A/G agarose beads (Invitrogen) for 1 h at 4 °C. The supernatants were incubated with 2 μg of anti-BIP antibody (CST) or normal IgG (Millipore, negative control) overnight at 4 °C. The next day, 20 μL of protein A/G beads were added and rotated for 2 h at 4 °C to capture the antibody‒protein complexes. The beads were washed with IP lysis buffer, and the bound proteins were eluted and analyzed by WB.

### Animal study

Animal experiments were approved by the Ethics Committee of Southwest Medical University (Approval No. swmu20230056) and conducted in accordance with the ARRIVE guidelines.^24^ A total of 30 four-week-old female BALB/c nude mice (SPF grade) were purchased from Hunan SJA Laboratory Animal Co. (Hunan, China). The mice were housed under controlled conditions (22 °C ± 2 °C, 12 h light/dark cycle) with free access to food and water. Stable knockdown of circRAB5A and BIP was achieved by lentivirus expressing shRNA targeting circRAB5A (sh-circRAB5A) and BIP (sh-BIP) and verified by qRT-PCR. CRC xenografts were established by subcutaneous injection of 5 × 10⁶ SW480 cells into the right axilla of the mice. When the tumor volume reached ~75–100 mm³ (7 d post-injection), mice were randomly assigned to five groups (*n* = 6 per group) using a random number table method. Prior to tumor irradiation, all the mice were anesthetized using a 5% isoflurane and oxygen mixture for rapid induction of anesthesia and maintained at 1.5%–2% isoflurane concentration during the irradiation procedure to induce a stable plane of anesthesia while minimizing respiratory and cardiovascular stress. Then, the mice received local tumor irradiation using an X-RAD 320 Biological Irradiator (Precision X-ray, USA) at a dose rate of 0.5 Gy/min. The mice in the sh-NC + radiation group received 3 Gy in 2 fractions (consecutive days), and those in the sh-BIP group received a single dose of 2 Gy. Tumor growth was monitored twice weekly using calipers. The tumor volume was calculated using the following formula: volume (mm³) = 0.5 × width² × length. Four weeks after tumor implantation, the mice were euthanized using an intraperitoneal injection of tribromoethanol (20 μL/g, AbMole, USA) following the cervical dislocation method. Last, tumors were harvested, weighed, and processed for subsequent analysis.

### Immunohistochemistry and TUNEL staining

Xenograft tumor tissues were fixed in 4% PFA, paraffin-embedded, and sectioned into 5-μm slides. The sections were then deparaffinized, rehydrated, and rinsed with PBS. After incubation with 3% H₂O₂ to block endogenous peroxidase activity, the slides were treated with a 5% BSA solution (Beyotime) for 30 min at room temperature. The primary antibody, rabbit anti-Ki67 (Abclonal), was diluted 1:200 with PBS, and the slides were incubated overnight at 4 °C. The next day, the secondary goat anti-rabbit HRP (Abclonal) was diluted 1:400 with PBS and incubated with the slides for 2 h at room temperature. A DAB staining kit (Abcam, USA) was used to develop a brown color. TUNEL staining was performed according to the manufacturer's protocol using a TUNEL staining kit (Beyotime Biotechnology). Finally, the nuclei were stained with hematoxylin, and images were acquired using an Olympus microscope (Japan).

### GEO analysis

Differentially expressed circRNAs in radioresistant CRC were identified using publicly available transcriptomic data from the GEO database (https://www.ncbi.nlm.nih.gov/geo/). The raw data of the GSE186940 dataset by Shao. Y. et al.^25^ were downloaded, preprocessed, and visualized using R 4.3.1. The thresholds of the differentially expressed circRNAs were set as: |log₂(fold change)| > 1, FDR < 0.05. Those with all zero expression count in the radiosensitive or radioresistant group were excluded.

### Statistical analyses

All experimental data were acquired from at least three individual replications. Statistical analyses were performed using GraphPad Prism 9.0 (GraphPad Software, San Diego, CA, USA) and R version 4.3.1. The data are presented as mean ± standard deviation (SD). For comparisons between two groups, Student's *t*-test was applied to normally distributed data with homogeneous variances, whereas the Mann‒Whitney U test was used for non-normally distributed or heteroscedastic data. For comparisons among three or more groups, one-way analysis of variance (ANOVA) followed by Tukey's honest significant difference (HSD) post-hoc test was applied. The correlation analysis was performed by GraphPad using a simple linear regression model. Overall survival was estimated using the Kaplan‒Meier method and compared between groups using the log-rank test. Statistical significance was defined as *p* < 0.05, with *p* < 0.01 and *p* < 0.001 indicating high significance.

## Results

### CircRAB5A is significantly downregulated in radioresistant colorectal cancer tissues and cell lines

**Figure 1.**
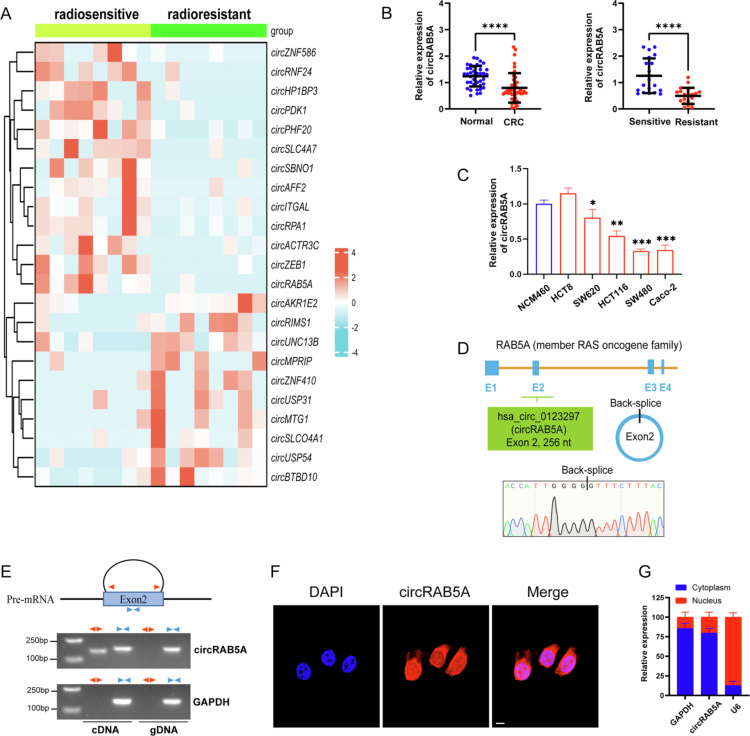
CircRAB5A was downregulated in radioresistant CRC. (A) The most differentially expressed circRNA from GSE186940 is depicted as a heat map. We focused on the remarkable downregulation of circRAB5A (hsa-circ-0123297) was focused. (B) Validation of circRAB5A expression in 40 pairs of clinical samples using qRT-PCR. Comparison of circRAB5A levels in radiosensitive and radioresistant CRC samples. (C) qRT-PCR results showing the expression of circRAB5A in multiple CRC cell lines (HCT8, SW620, HCT116, SW480, and Caco-2) and the normal colon epithelial cell line NCM460. CircRAB5A expression was decreased in most CRC cell lines. (D) Design and verification of circRAB5A primers. Divergent primers amplified the back-splice site of circRAB5A and the sequences were verified by sequencing. (E) Amplification of circRAB5A by divergent primers in cDNA but not gDNA. (F) Results of circRAB5A FISH staining. Scale bar = 5 μm. (G) Results of the sub-cellular fraction assay; GAPDH represents the cytoplasmic fractions, and U6 represents the nuclear fraction. *p < 0.05; **p < 0.01; ***p < 0.001; ****p < 0.0001.

To identify circRNAs associated with radioresistance, we analyzed the GEO dataset GSE186940, which includes the transcriptomic profiles of eight radiosensitive and eight radioresistant CRC patient tissues. The screening criteria was set as follows: (1) differential expression threshold (|log₂(fold change)| > 1, FDR < 0.05). (2) The original expression count were not all zero. (3) possessing typical circRNA characteristics: sequences available in circBase and suitable lengths (200–500 nt), and forming by exon back-splicing. (4) Not reported. Based on that, hsa-circ-0123297 (designated circRAB5A) was focused, which was the most significantly downregulated circRNA in radioresistant samples (Figure 1A). To validate this finding, we examined circRAB5A expression in 40 pairs of clinical CRC tissue samples (20 radiosensitive, 20 radioresistant, and adjacent normal mucosal tissues). qRT-PCR confirmed that circRAB5A levels were significantly reduced in CRC tissues compared to adjacent normal tissues and further decreased in radioresistant samples compared with radiosensitive CRC samples (Figure 1B). These results were consistent with the GEO dataset showing that circRAB5A was downregulated in radioresistant CRC tissues.

Next, we assessed circRAB5A expression in CRC cell lines. qRT-PCR showed that circRAB5A was downregulated in CRC cell lines (SW480, SW620, HCT116, and Caco-2) compared to that in the normal colon epithelial cell line NCM460 (Figure 1C). To confirm its circular nature, we performed divergent primer PCR, in which divergent primers amplified the back-splice junction in cDNA but not in genomic DNA (gDNA), and Sanger sequencing verified the back-splice sequence (Figure 1D and E). Subcellular fractionation and FISH assays demonstrated that circRAB5A was predominantly localized to the cytoplasm (Figure 1F and G). ACT-D treatment revealed that circRAB5A had a longer half-life than linear RAB5A (Supplementary Figure S1A), confirming its stability. RNase R digestion assays showed that circRAB5A was resistant to exonuclease degradation, unlike linear RAB5A mRNA (Supplementary Figure S1B). Additionally, circRAB5A was not reverse-transcribed by oligo (dT) primers, indicating a non-polyadenylated structure (Supplementary Figure S1C). Collectively, these data confirm that circRAB5A is a stable, cytoplasm-enriched circular RNA that was first identified to be downregulated in radioresistant CRC.

### ADAR1 suppresses circRAB5A biogenesis via Alu Jo/Jr element binding

**Figure 2.**
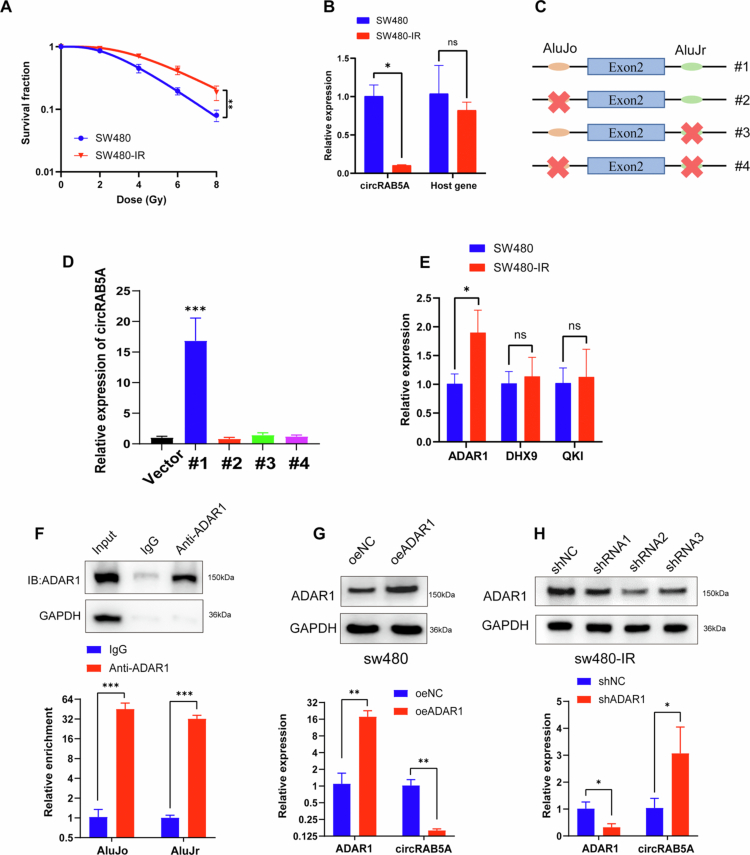
ADAR1 suppresses circRAB5A biogenesis via binding intron Alu Jo/Jr binding. (A) Clonogenic survival assay verified the establishment of radiation-resistant SW480 cells (SW480-IR). (B) Comparison of circRAB5A and RAB5A mRNA levels in parental SW480 and SW480-IR cells. CircRAB5A was significantly downregulated in SW480-IR cells. (C) Sketch map of full-length or Alu Jo/Jr truncated expression vectors. (D) qRT-PCR results show that only the co-existence of Alu Jo/Jr significantly promoted the biogenesis of circRAB5A. (E) qRT-PCR results of ADAR1, DHX9, and QKI expression in parental SW480 cells and SW480-IR cells. ADAR1 expression significantly increased in SW480-IR cells. (F) RIP results using the anti-ADAR1 antibody. The ADAR1 antibody significantly enriched Alu Jo/Jr sequences compared to control IgG. (G) qRT-PCR and WB results showed that the overexpression of ADAR1 in SW480 cells significantly decreased circRAB5A expression. (H) qRT-PCR and WB results showed that ADAR1 knockdown increased circRAB5A expression in SW480-IR cells. Ns, non-significance; *p < 0.05; **p < 0.01; ***p < 0.001.

To investigate the mechanism underlying circRAB5A downregulation in radioresistance, we established radiation-resistant SW480 cells (SW480-IR) via 10 fractions of 2 Gy irradiation (total 20 Gy over 5 weeks), which was validated by a clonogenic survival assay (Figure 2A and Supplementary Figure S1D). qRT-PCR confirmed that circRAB5A levels were remarkably lower in SW480-IR cells than in parental SW480 cells, whereas linear RAB5A mRNA expression remained unchanged (Figure 2B), indicating reduced back-splicing efficiency rather than decreased host gene transcription.

Next, to elucidate the molecular mechanism underlying circRAB5A downregulation, we performed a mechanistic assay. Given that the conserved Alu sequences in the flanking introns were responsible for circRNA formation, we explored the Alu Jo and Alu Jr elements around the circRAB5A linear sequence and whether they participate in circRAB5A biogenesis. We constructed expression vectors containing full-length RAB5A pre-mRNA or Alu Jo/Jr-truncated variants (Figure 2C). qRT-PCR revealed that only the full-length vector (containing both Jo and Jr) significantly increased circRAB5A biogenesis (Figure 2D). Then, via catRAPID online software, the results predicted that the RBPs ADAR1 and DHX9 could interact with both the Alu Jo and Jr sequences (Supplementary Tables S3 and S4). First, we performed a qRT-PCR to explore whether these RBPs expressions were altered during radiation, including ADAR1, DHX9 and QKI (negative control). The results showed only ADAR1 expression was significantly upregulated in SW480-IR cells, whereas the DHX9 and QKI levels were unchanged (Figure 2E). Second, to test whether ADAR1 modulates circRAB5A via Alu Jo/Jr, we performed RNA immunoprecipitation (RIP) and found that anti-ADAR1 antibodies significantly enriched Alu Jo/Jr sequences compared to the IgG control (Figure 2F), confirming direct binding. The enforced expression of ADAR1 in parental SW480 cells reduced circRAB5A levels (Figure 2G), whereas ADAR1 knockdown in SW480-IR cells restored circRAB5A expression (Figure 2H). Finally, to elucidate the relation between ADAR1 and circRAB5A in CRC tissues, we also validated the expression of ADAR1 in 20 radiosensitive and 20 radioresistant CRC samples. The PCR result displayed despite ADAR1 was upregulated in the radioresistant group (Supplementary Figure S1E), the correlation analysis did not show a significant relation between ADAR1 and circRAB5A in CRC samples (Supplementary Figure S1F; *R* = −0.173, *p* = 0.283). It indicates ADAR1 probably was not the exclusive RBP that regulates circRAB5A biogenesis. Collectively, these data demonstrate that ADAR1 upregulation in radioresistant CRC suppresses circRAB5A biogenesis by binding to Alu Jo/Jr elements, thereby reducing back-splicing efficiency.

### CircRAB5A depletion confers radioresistance by promoting protective autophagy and inhibiting apoptosis

**Figure 3.**
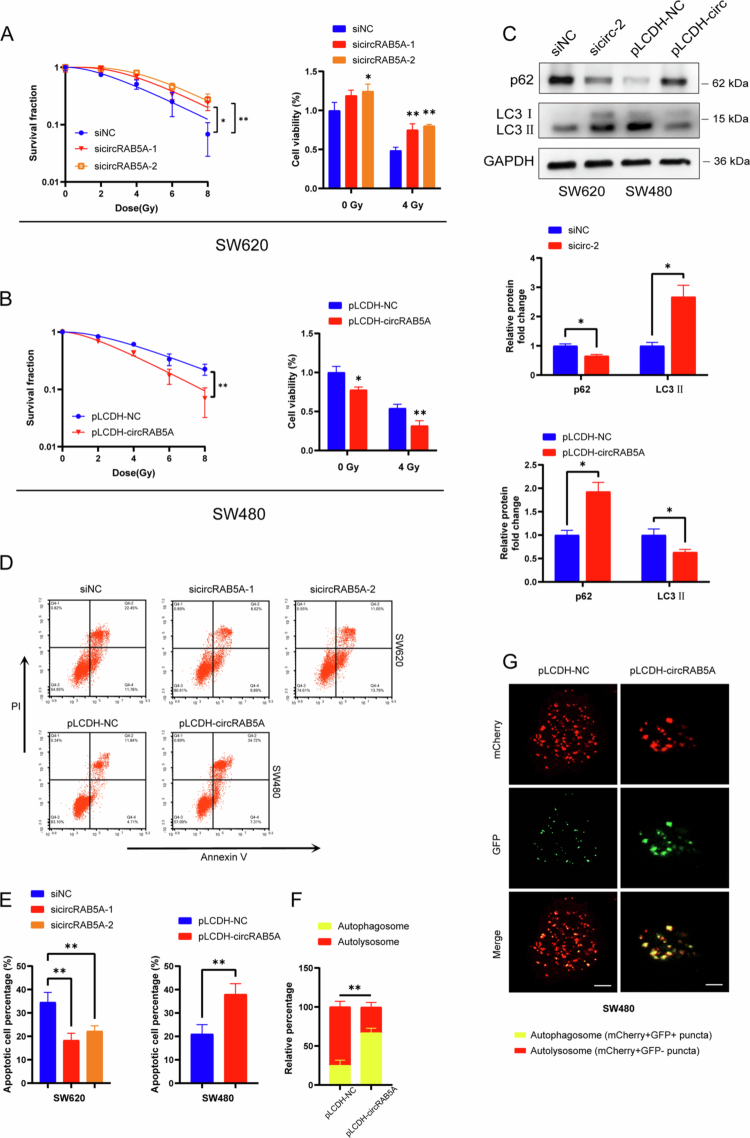
CircRAB5A depletion confers radioresistance of CRC cells by regulating the autophagy-apoptosis balance. (A) Clonogenic assay results in SW620 cells showed that downregulation of circRAB5A significantly increased the radiosensitivity of SW620 cells, and that cell viability increased at a treatment dose of 4 Gy. (B) Clonogenic assay results in SW480 cells showed that upregulation of circRAB5A decreased the radiosensitivity of SW480 cells, and that cell viability at a treatment dose of 4 Gy was decreased. (C) WB results showed that forced expression of circRAB5A decreased LC3-II expression and promoted p62 expression. Knockdown of circRAB5A led to the opposite result. (D) Annexin V/PI double-staining results showed that after radiation treatment, the percentage of apoptotic cells significantly decreased with the reduction of circRAB5A. (E) The statistical analysis of the Annexin V/PI double-staining results. (F) The statistical analysis of mCherry-GFP-LC3 dual-fluorescence reporters. (G) Forced expression of circRAB5A decreases autolysosome formation (mCherry+GFP− puncta) using mCherry-GFP-LC3 dual-fluorescence reporters. *p < 0.05; **p < 0.01; ***p < 0.001.

To determine the functional role of circRAB5A in radioresistance, we performed clonogenic survival assays using CRC cells with altered circRAB5A expression. In SW620 cells, transfection with sicircRAB5A (knockdown efficiency > 50%, Supplementary Figure S2A) significantly increased colony survival from to 0 to 8 Gy and elevated cell viability at 4 Gy (Figure 3A and Supplementary Figure S2C). Conversely, enforced expression of circRAB5A in SW480 cells reduced colony survival, reduced cell viability at 4 Gy, and sensitized cells to radiation (Figure 3B and Supplementary Figure S2B and D). These results confirmed that circRAB5A negatively regulates radioresistance in CRC cells. Next, we assessed apoptosis by Annexin V/PI staining. After 4 Gy radiation, sicircRAB5A-transfected SW620 cells showed a significant reduction in apoptotic cells, while circRAB5A-overexpressing SW480 cells exhibited an increase in apoptosis (Figure 3D and E).

Given the role of autophagy in maintaining intracellular homeostasis in response to various stimuli, we evaluated the protective role of autophagy in the radioresistance of CRC cells and the underlying mechanism of circRAB5A. We treated SW480 cells with the autophagy inhibitor chloroquine (CQ, 10 μM), and alterations in LC3-II and p62 proteins were analyzed by WB (Supplementary Figure S3D). Furthermore, the results showed that CQ remarkably decreased cell viability and increased apoptotic cells after radiation treatment compared with the control group (DMSO) (Supplementary Figure S3A–C), confirming the protective role of autophagy against radiation in CRC cells. Next, we evaluated autophagic flux by using WB and mCherry-GFP-LC3 dual-fluorescence reporters. In SW620 cells, sicircRAB5A decreased p62 and increased LC3-II (Figure 3C). On the contrary, in SW480 cells, circRAB5A overexpression reduced LC3-II expression, indicating an autophagy inhibition, which was further confirmed by reduced autolysosome formation (mCherry+GFP− puncta) (Figure 3F and G). To further reveal the role of circRAB5A in regulating autophagy and its mechanistic link with radioresistance, we performed a functional assay using CQ or rapamycin (Rapa, an autophagy inducer, 25 nM) upon circRAB5A overexpression or knocking down. The CCK8 and apoptosis assay demonstrated that CQ abolished sicircRAB5A-induced radioresistance while Rapa counterbalanced the decreased radioresistance induced by forced circRAB5A expression (Supplementary Figure S4). Taken together, these data indicate that circRAB5A depletion enhances radioresistance via promoting protective autophagy and suppressing apoptosis.

### CircRAB5A regulates BIP protein stability through TRIM21-mediated ubiquitination

**Figure 4.**
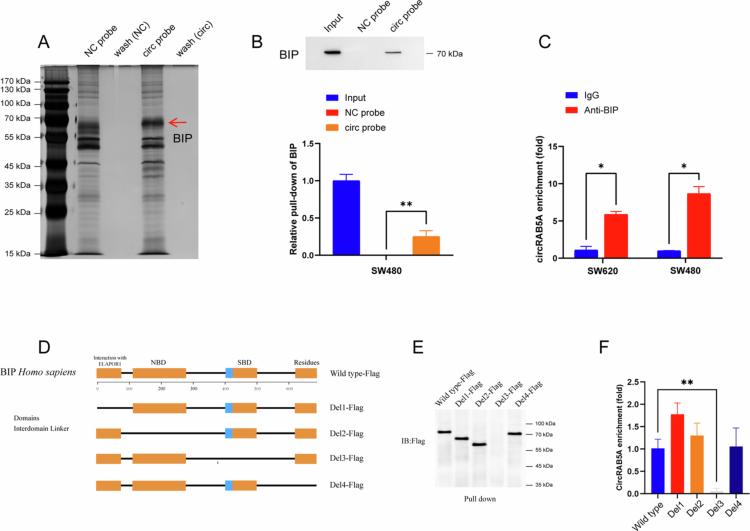
CircRAB5A interacted with ER chaperonin, BIP in CRC cells. (A) Silver staining results of the RNA pull-down assay using circRAB5A probes. Differentially enriched bands in the circ group were detected at a molecular weight of 70 kDa. (B) Verification of BIP protein enrichment in the circ group via WB detection. (C) RIP results using an anti-BIP antibody. In the BIP-bound extract, the enrichment of circRAB5A was significantly higher than that of the control IgG, indicating an interaction between the circRAB5A and BIP proteins in CRC cells. (D) Sketch map of wild-type or truncated BIP expression vectors. (E) RNA pull-down assay using circRAB5A probes following WB of Flag antibody showed circRAB5A probe failed to pull-down Del3 truncated protein. (F) RIP results using an anti-Flag antibody. Del3 truncated protein could not precipitated circRAB5A. *p < 0.05; **p < 0.01.

To investigate the molecular mechanism of circRAB5A, we performed RNA pull-down assays using biotinylated circRAB5A probes. Silver staining of the eluted proteins revealed a prominent band at approximately 70 kDa in the circRAB5A group (Figure 4A), which was identified as BIP (GRP78) by mass spectrometry (10 unique peptides, sequence coverage 22%). Western blotting confirmed that BIP was specifically enriched in circRAB5A pull-downs (Figure 4B). Reciprocal RNA immunoprecipitation (RIP) with anti-BIP antibodies showed that circRAB5A was significantly enriched in BIP-bound fractions vs. the IgG control (Figure 4C), confirming the direct interaction. To clarify the molecular basis of the circRAB5A-BIP interaction, a truncation experiment was conducted. As shown in Figure 4D, we divided the BIP protein into four main domains and constructed wild-type (full-length) and individually truncated proteins into expression vectors. The result showed circRAB5A probe failed to pull-down Del3 truncated protein (lacking an internal linker and SBD domain, Position: 409–500) (Figure 4E). Also, RIP with an anti-Flag antibody could not precipitate circRAB5A in the Del3 group (Figure 4F). It indicates circRAB5A interacts with the internal linker and SBD domain of the BIP protein.

**Figure 5.**
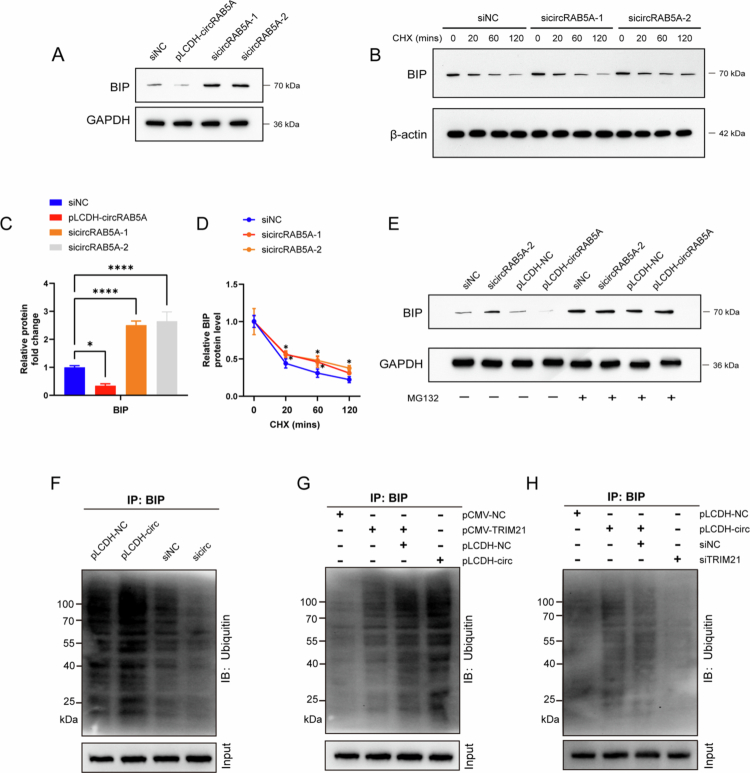
CircRAB5A regulates BIP protein stability through TRIM21-mediated ubiquitination. (A, C) WB results of BIP protein levels after circRAB5A overexpression or knockdown. circRAB5A depletion upregulated and circRAB5A forced expression downregulated BIP protein levels. (B, D) CHX chase assay results showed that circRAB5A depletion significantly prolonged the stability of BIP protein. The statistical analysis was depicted in the line chart. (E) Rescue experiments in SW480 cells showed that the degradation of BIP induced by circRAB5A overexpression was rescued after treatment with the proteasome inhibitor MG132. (F) Ubiquitination assay results showed that circRAB5A knockdown decreased, while forced expression of circRAB5A increased, the ubiquitination of BIP. (G, H) Ubiquitination assay results showed that enforced TRIM21 expression in SW480 cells enhanced BIP ubiquitination, which was further increased by circRAB5A co-overexpression. In contrast, TRIM21 knockdown abrogated circRAB5A-induced BIP ubiquitination. *p < 0.05; ****p < 0.0001.

Next, we examined the effects of circRAB5A on BIP expression. WB showed that circRAB5A overexpression reduced BIP protein levels, while sicircRAB5A increased BIP (Figure 5A and B). To test whether this regulation occurs at the post-translational level, we performed cycloheximide (CHX) chase assays, and the results showed that the half-life of BIP was prolonged in sicircRAB5A cells and shortened in circRAB5A-overexpressing cells (Figure 5C and D), indicating that circRAB5A destabilizes BIP. To determine whether the ubiquitin-proteasome pathway mediates BIP degradation, we treated cells with the proteasome inhibitor MG132 (10 μM, 6 h). MG132 rescued BIP downregulation in circRAB5A-overexpressing cells (Figure 5E), thus confirming proteasomal degradation. Ubiquitination assays showed that circRAB5A overexpression increased BIP ubiquitination, whereas sicircRAB5A reduced it (Figure 5F). Given that TRIM21 is an E3 ubiquitin ligase of BIP,^26^ we tested its role. The enforced expression of TRIM21 in SW480 cells enhanced BIP ubiquitination (Supplementary Figure S5B), which was further increased by circRAB5A co-overexpression (Figure 5G). Conversely, TRIM21 knockdown (siTRIM21, efficiency > 75%, Supplementary Figure S5A) abrogated circRAB5A-induced BIP ubiquitination (Figure 5H). These data confirmed that circRAB5A promotes TRIM21-mediated BIP ubiquitination, thereby reducing BIP stability.

### The circRAB5A/BIP axis modulates radiation-induced autophagy-apoptosis balance via the p-Akt/Beclin1 pathway

**Figure 6.**
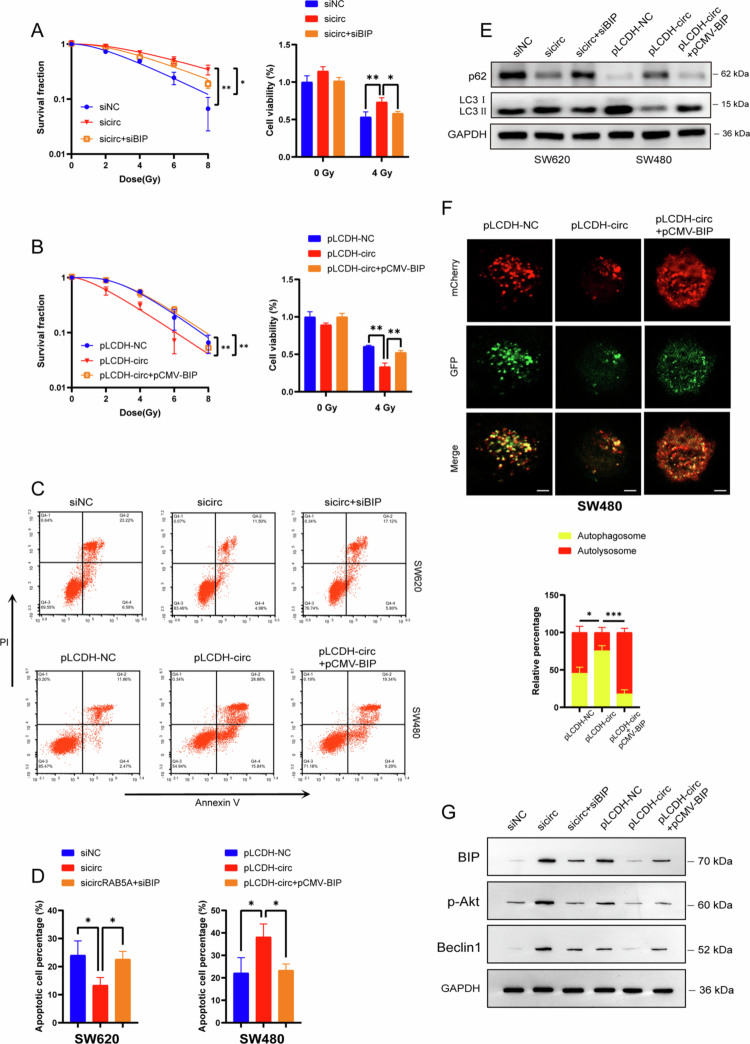
The circRAB5A/BIP axis modulates autophagy-apoptosis balance in CRC cells via the p-Akt/Beclin1 pathway. (A) Results of the co-transfection experiments in CRC cells. Co-transfection of circRAB5A siRNA with BIP siRNA abolished the sicircRAB5A-induced increase in colony survival and cell viability. (B) Co-transfection with BIP reversed the circRAB5A-induced radiosensitive phenotype. (C, D) Annexin V/PI apoptosis assay results showed an increase in apoptotic cells by sicircRAB5A, and the decrease in apoptotic cells by circRAB5A forced expression was reversed by siBIP and BIP overexpression, respectively. (E, F) WB detection of LC3 and p62, and mCherry-GFP-LC3 dual-fluorescence reporters consistently indicated that the expression of BIP counterbalanced circRAB5A-mediated inhibition of autophagy. (G) WB blotting demonstrated that the circRAB5A/BIP axis conferred radioresistance in CRC cells by regulating the p-Akt/Beclin1 signaling pathway. *p < 0.05; **p < 0.01; ***p < 0.001.

To validate the functional link between circRAB5A and BIP in radioresistance, we performed rescue experiments by co-transfecting SW620 cells with sicircRAB5A and siBIP (BIP knockdown efficiency > 65%, Supplementary Figure S5C). Clonogenic survival assays showed that the co-knockdown of BIP abrogated the radioresistance induced by sicircRAB5A and decreased cell viability at 4 Gy (Figure 6A and Supplementary Figure S6A). Conversely, the overexpression of BIP in circRAB5A-overexpressing SW480 cells restored radioresistance and cell viability at 4 Gy (Figure 6B and Supplementary Figures S5D and S6B). These results confirmed that BIP is a critical downstream effector of circRAB5A in regulating radioresistance.

Next, we assessed the effect of the circRAB5A/BIP axis on apoptosis and autophagy. Annexin V/PI staining revealed that the sicircRAB5A-induced reduction in apoptosis was reversed by siBIP, while BIP overexpression attenuated the increase in apoptosis caused by circRAB5A overexpression (Figure 6C and D). Western blotting showed that sicircRAB5A increased LC3-II expression and decreased p62 levels, which were abolished by siBIP (Figure 6E). Conversely, BIP overexpression restored autophagic flux in circRAB5A-overexpressing cells (Supplementary Figure S6C and D). mCherry-GFP-LC3 imaging confirmed these findings. The percentage of autolysosomes was reduced by circRAB5A overexpression but restored by BIP co-overexpression (Figure 6F).

To identify the signaling pathway mediating this crosstalk, we examined the p-Akt/Beclin1 axis, a key regulator of autophagy-apoptosis balance. Western blotting showed that circRAB5A overexpression decreased p-Akt and Beclin1 levels, while sicircRAB5A increased p-Akt and Beclin1 levels (Figure 6G). BIP overexpression rescued p-Akt and Beclin1 levels in circRAB5A-overexpressing cells, and BIP knockdown reversed sicircRAB5A-induced upregulation of p-Akt/Beclin1. Collectively, these data demonstrated that the circRAB5A/BIP axis modulates radioresistance by regulating the p-Akt/Beclin1 pathway, which coordinates protective autophagy and apoptosis in CRC cells.

### The circRAB5A/BIP axis promotes radioresistance in CRC xenograft models

**Figure 7.**
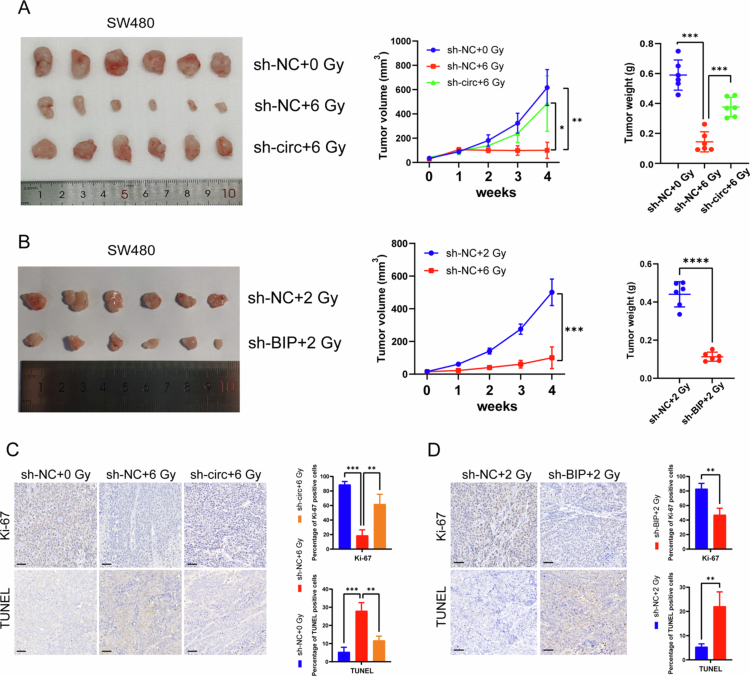
*In vivo* experiments revealed the role of circRAB5A/BIP axis on radioresistance. (A) *In vivo* experimental results obtained using a subcutaneous xenograft model of BALB/c nude mice. Stable knockdown of circRAB5A attenuated the effect of radiation treatment and enhanced radioresistance, as shown by changes in tumor volume and weight. (B) Effect of stable BIP knockdown on sensitivity to radiotherapy. Sh-BIP sensitized CRC cells to radiotherapy even at a low dose of radiation (2 Gy). (C, D) Immunohistochemistry (ICC) of Ki67 and TUNEL staining results showed that circRAB5A knockdown protected cell proliferation and inhibited apoptosis under radiation. However, sh-BIP yielded the opposite results. *p < 0.05; **p < 0.01; ***p < 0.001; ****p < 0.0001.

To validate the above findings *in vivo*, we established subcutaneous xenograft models using SW480 cells stably expressing shRNA targeting circRAB5A (sh-circRAB5A), BIP (sh-BIP), or a non-targeting control (sh-NC) as the control group. Tumor growth was monitored weekly, and radiotherapy was initiated when the tumors reached 75–100 mm³ (day 7 post-injection). In sh-NC control mice, local irradiation (3 Gy × 2 fractions) significantly reduced the tumor volume and weight (Figure 7A). However, in the sh-circRAB5A group, irradiation had a minimal effect as the tumor volume and tumor weight were higher than those in the irradiated sh-NC group, but showed no significant differences when compared with the non-irradiated sh-NC group.

Histological analysis confirmed these results TUNEL staining revealed a reduction in apoptotic cells in sh-circRAB5A tumors vs. sh-NC tumors after irradiation, while Ki67 staining showed an increase in proliferating cells (Figure 7C). Western blotting of tumor lysates demonstrated that sh-circRAB5A increased BIP levels and LC3-II expression, which was consistent with enhanced protective autophagy (Supplementary Figure S7).

To test the therapeutic potential of targeting BIP, sh-BIP mice were treated with a low radiation dose (2 Gy × 1 fraction). Sh-BIP tumors showed a significant reduction in tumor volume and weight compared with sh-NC mice (Figure 7B). Histological analysis of TUNEL and Ki67 staining also showed that the percentage of apoptotic and proliferating cells changed consistently with phenotype (Figure 7D). Collectively, these *in vivo* data demonstrate that the circRAB5A/BIP axis promotes radioresistance by enhancing protective autophagy and inhibiting apoptosis and that targeting BIP sensitizes CRC tumors to radiotherapy.

## Discussion

Radiotherapy has attracted considerable attention in CRC treatments in recent years; however, radioresistance remains a major clinical challenge, and its regulatory mechanisms remain unclear. The role of circRNAs, novel regulatory RNA molecules, has attracted interest in recent years.^27^ The differential expression of certain circRNAs is associated with acquired resistance to radiotherapy.^28^ However, radioresistance-related circRNAs and their mechanisms have not been elucidated in CRC. Here, we identified a novel molecular axis “ADAR1/circRAB5A/BIP” that governs radioresistance by coordinating protective autophagy and apoptosis. Our findings provide critical insights into the mechanisms underlying radiation adaptation in CRC and highlight potential therapeutic targets for enhancing treatment efficacy.

First, we demonstrated that circRAB5A is a previously uncharacterized, cytoplasm-enriched circular RNA that is significantly downregulated in radioresistant CRC tissues and cell lines. In the 40 pairs of collected clinical samples, downregulation of circRAB5A was also observed, indicating that the decrease in circRAB5A might be a biomarker associated with radioresistance. This finding is expected to fill a gap in the literature. For example, Lee et al. reported that the LRP-1 protein is a radioresistant marker^29^ and Munakata et al. demonstrated that the expression of SCGB2A1 was a prognostic marker for CRC associated with radioresistance.^30^ Further exploration of our work will provide new evidence that circRNAs are biomarkers associated with radioresistance and prognosis in CRC patients.^31^ Together, it will provide a radioresistant-related biomarker network that is available for the clinical use and evaluation of radio-responses.^32^

Next, we elucidated that the downregulation of circRAB5A was not due to reduced transcription of its host gene RAB5A but rather impaired back-splicing efficacy, driven by ADAR1 upregulation in radioresistant CRC cells. ADAR1, an RNA-editing enzyme, binds Alu Jo/Jr elements in the flanking introns of the circRAB5A locus, thereby suppressing its biogenesis. This mechanism expands our understanding of ADAR1's role in cancer, and the aberrant upregulation of ADAR1 contributes greatly to carcinogenesis and cancer progression from multiple aspects.^33^ Although prior studies have focused on its regulation of mRNA splicing^34^ or miRNA targeting,^35^ our work revealed a new layer of regulation in which ADAR1 modulates circRNA biogenesis via Alu elements, a pathway by which ADAR1 could regulate radioresistance by modulating key circRNA expression.^36^

Functionally, circRAB5A depletion confers radioresistance by promoting protective autophagy and inhibiting apoptosis. The balance between autophagy and apoptosis is a critical determinant of the cellular response to radiation.^37^ In CRC cells, over-activation of autophagy showed strong protective effects against radioresistance. This aligns with the emerging evidence that autophagy acts as a survival mechanism under radiation stress. During this process, the stability of the BIP protein is crucial because sustained BIP expression promotes autophagy and leads to resistance. In our study, we demonstrated that circRAB5A directly interacts with BIP, an ER chaperone that modulates proteostasis. By interacting with the internal linker and SBD domain of the BIP protein, circRAB5A enhances TRIM21-mediated ubiquitination and destabilizes the BIP protein, thereby downregulating the BIP-mediated p-Akt/Beclin1 signaling pathway, which regulates autophagy-apoptosis crosstalk. Our results demonstrated that the circRAB5A/BIP axis tilted the autophagy-apoptosis balance toward autophagic survival, which led to enhanced radioresistance in CRC. This finding expands the understanding of the autophagy-apoptosis balance during the formation of radioresistance in CRC and proves the significance of circRNA in this process.

Notably, in recent years, the targeted delivery of non-coding RNAs, especially circRNAs, has shown promising therapeutic effects, as circRNAs are more stable and participate in signal transduction from multiple aspects.^38^ ^,^ ^39^ For example, Ding et al. reported that circRERE-AAV delivery significantly inhibited CRC tumor growth via the miR-6837-3p/MAVS axis, thereby activating type I IFN signaling. Combination treatment with circRERE-AAV and anti-PD-1 antibodies also exhibited synergistic effects.^40^ Wang et al. reported that exosome-delivered si-ciRS-122 into drug-resistant CRC cells could suppress glycolysis and alleviate resistance to oxaliplatin via modulating “ciRS-122/miR-122/PKM2” pathway.^41^ In our study, when we knocked down circRAB5A expression in mice xenograft model, we observed a significant increase in radioresistance in CRC tumors. This indicates that targeted delivery of circRAB5A sensitizes CRC cells to radiotherapy and inhibits acquired radioresistance. Additionally, our *in vivo* data also supported that BIP knockdown enhanced the radiosensitivity of CRC xenografts, even at low doses. This indicates that BIP proteins also have potential as therapeutic anti-tumor targets.^42^ ^,^ ^43^ These findings are also particularly relevant in combination with immunotherapy, as ER stress and autophagy have been linked to immune evasion in CRC.^44-46^ Further exploration of our work will reveal more functions of circRAB5A and highlight its significance in anti-tumor therapy.

This study has several limitations that warrant further investigation. First, we validated the “ADAR1/circRAB5A/BIP” axis in SW480 and SW620 cell lines; however, in other CRC subtypes, such as microsatellite instability-high (MSI-H) or KRAS-mutated tumors, the function and generalizability of this axis are currently under investigation. Second, our animal models used subcutaneous xenografts, which lack the tumor microenvironment of primary CRC. Third, we utilized a circRAB5A knocking down model in an animal study. Considering the endogenous low level of circRAB5A, a circRAB5A overexpression model would provide more meaningful insight. Future studies in orthotopic or transgenic mouse models would better recapitulate disease biology. A circRAB5A overexpression model would be better. Additionally, the current clinical sample size was limited, and the follow-up duration was short. We failed to fully elucidate the clinical significance of circRAB5A and revealed its correlation with prognosis. To address these concerns, a large cohort study of CRC patients with a long-term follow-up was designed and conducted.

In summary, our study defines a novel regulatory circuit, “ADAR1/circRAB5A/BIP”, that governs CRC radioresistance by coordinating autophagy and apoptosis. We identified circRAB5A as a key regulator of this process. Mechanistically, we found that circRAB5A directly interacts with the internal linker and SBD domain of the BIP protein, an ER chaperone protein for proteostasis. By enhancing TRIM21-mediated ubiquitination, circRAB5A destabilizes the BIP protein, thereby downregulating the BIP-mediated p-Akt/Beclin1 signaling pathway, which modulates autophagy-apoptosis crosstalk. This extended the work of Wang et al.^26^ on BIP regulation by identifying a non-coding RNA (circRAB5A) as a novel upstream modulator of BIP stability. This axis provides a mechanistic framework for understanding the initiation of acquired radioresistance and identifying potential therapeutic targets to enhance radiotherapy efficacy. It also provides evidence for the targeted delivery of circRANs as an anti-tumor strategy.

## Supplementary Material

Supplementary material Supplementary Figure S2.docx

Supplementary material Supplementary Figure S3.docx

Supplementary material Supplementary Figure S1.docx

Supplementary material Supplementary Figure S4.docx

Figure Legends

Supplementary material Supplementary Tables.docx

Supplementary material Supplementary Figure S6.docx

Supplementary material Supplementary Figure S7.docx

Supplementary Figure S5.docx

Supplementary material ARRIVE Checklist

## Data Availability

The data involved in this article and supplementary materials are available from the corresponding author upon reasonable request. The supplementary tables and online tables for review are available at online repository (https://doi.org/10.6084/m9.figshare.30814766).
