# Supplementary material for: ADAR1-circRAB5A-BIP axis governs radiotherapy resistance in colorectal cancer through coordinating protective autophagy and apoptosis
Source: Cancer Biol Ther. 2026 Jun 21;27(1):2677975. doi: 10.1080/15384047.2026.2677975 (PMC13285610; doi:10.1080/15384047.2026.2677975)
Supplement: Supplementary material — Supplementary Figure S2.docx [file KCBT_A_2677975_SM6918.docx]

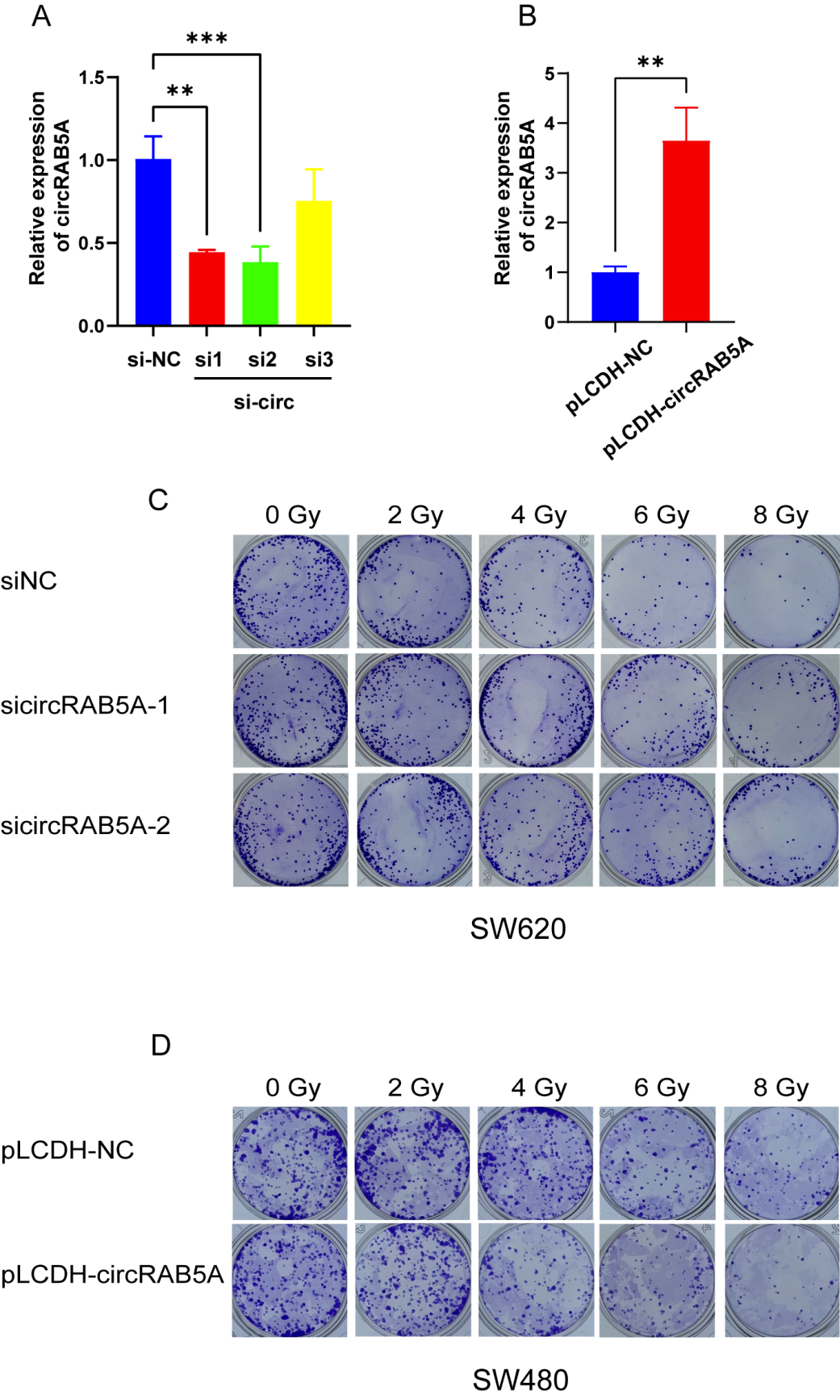


Supplementary Figure S2. Validation of knockdown and overexpression efficiency of circRAB5A vectors.

A. qRT-PCR results showed that sicircRAB5A efficiently knocked down circRAB5A expression by >50% in SW620 cells.

B. qRT-PCR results showed that pLCDH-circRAB5A induced a over 3-folds increase of circRAB5A in SW480 cells.

C, D: The original images of clonogenic survival assay.

**, P* < 0.05; **, *P* < 0.01; ***, *P* < 0.001.
