# Supplementary material for: ADAR1-circRAB5A-BIP axis governs radiotherapy resistance in colorectal cancer through coordinating protective autophagy and apoptosis
Source: Cancer Biol Ther. 2026 Jun 21;27(1):2677975. doi: 10.1080/15384047.2026.2677975 (PMC13285610; doi:10.1080/15384047.2026.2677975)
Supplement: Supplementary material — Supplementary Figure S3.docx [file KCBT_A_2677975_SM6919.docx]

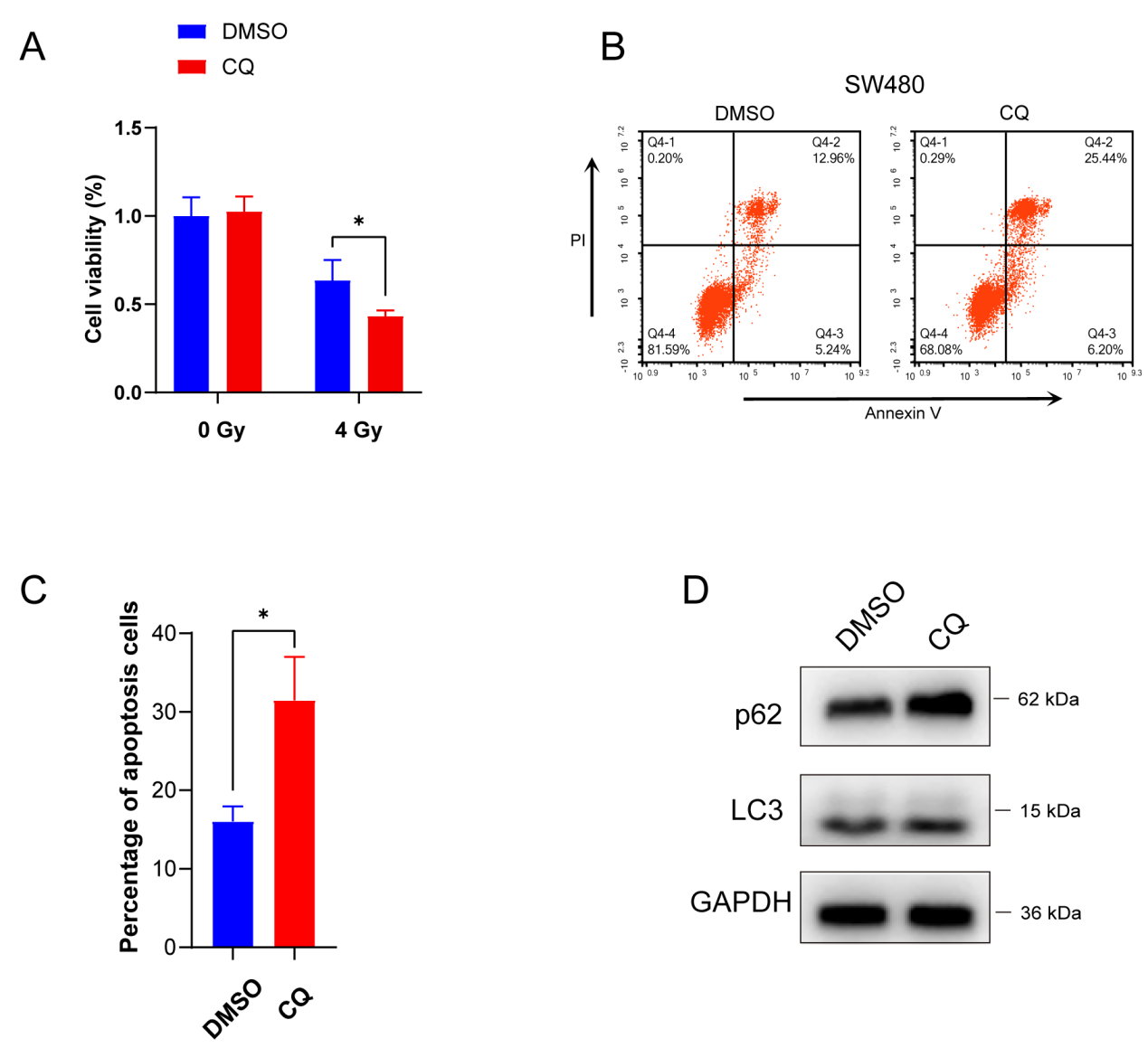


Supplementary Figure S3. Protective role of autophagy in radioresistance of CRC cells.

A. Cell viability results showed that CQ remarkably decreased cell viability after radiation treatment, compared with the control group (DMSO).

B. Apoptosis assay results showed that CQ increased apoptotic cells after radiation treatment, compared with the control group.

C. The statistical analysis of apoptosis assay.

D. Western blot of LC3-II and P62 showed that CQ treatment increased LC3-II expression and decreased P62 expression, indicating enhanced autophagic flux.

**, P* < 0.05
