# Supplementary material for: ADAR1-circRAB5A-BIP axis governs radiotherapy resistance in colorectal cancer through coordinating protective autophagy and apoptosis
Source: Cancer Biol Ther. 2026 Jun 21;27(1):2677975. doi: 10.1080/15384047.2026.2677975 (PMC13285610; doi:10.1080/15384047.2026.2677975)
Supplement: Supplementary material — Supplementary Figure S1.docx [file KCBT_A_2677975_SM6920.docx]

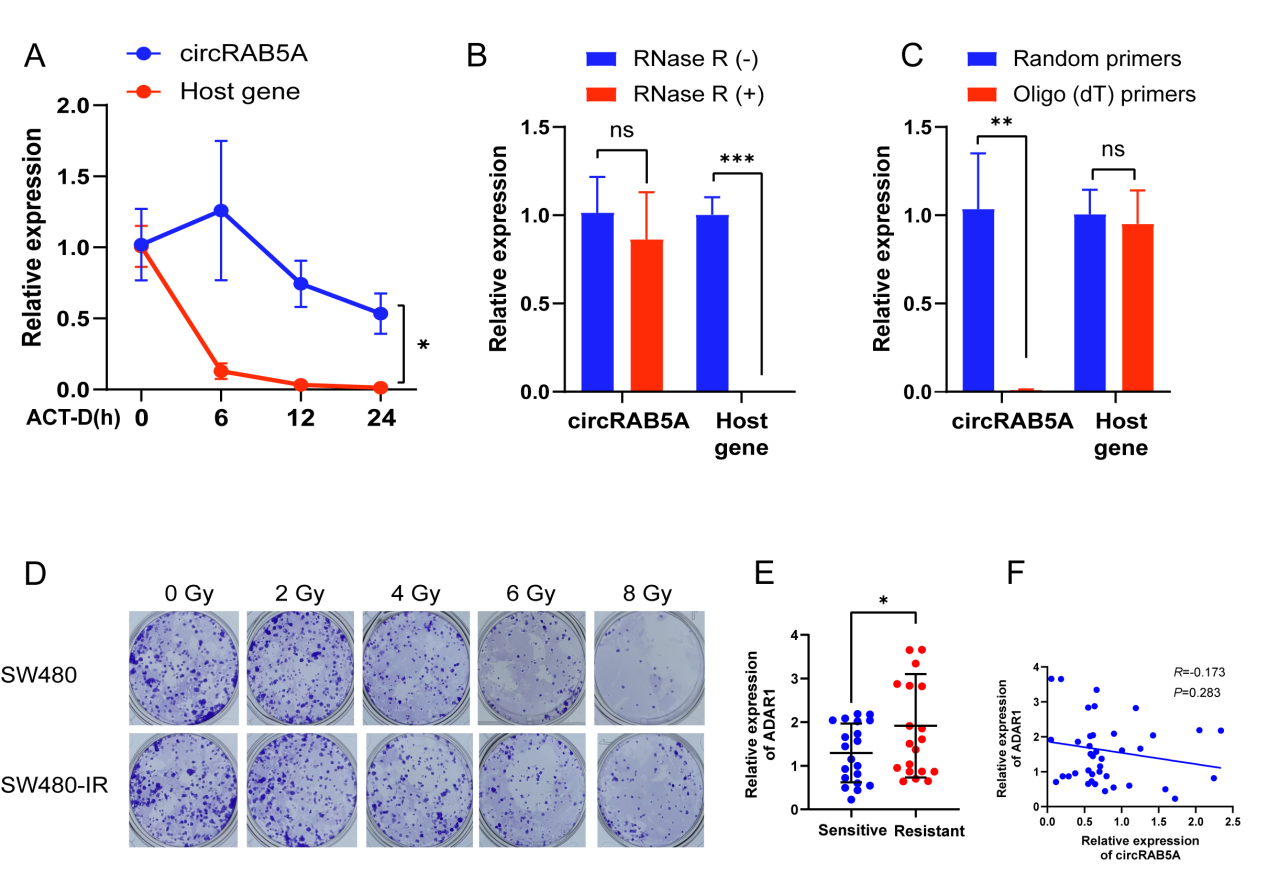


Supplementary Figure S1. Characterization of circRAB5A.

A: Cytoplasmic and nuclear fractions were isolated from SW480 cells. GAPDH was used as a cytoplasmic marker, and U6 was used as a nuclear marker. The results showed that circRAB5A was predominantly localized in the cytoplasm.

B: Total RNA from SW480 cells was treated with RNase R or mock digested. qRT-PCR results showed that circRAB5A was resistant to RNase R digestion, while linear RAB5A mRNA was degraded.

C: Total RNA from SW480 cells was reverse-transcribed using random primers or Oligo(dT) primers. qRT-PCR results showed that circRAB5A was not reverse-transcribed by Oligo(dT) primers, indicating a non-polyadenylated structure.

D: The original images of clonogenic survival assay.

E: The qPCR validated the expression of ADAR1 in 20 radiosensitive and 20 radioresistant CRC samples. The result displayedADAR1 was upregulated in radioresistant group.

F: The correlation analysis did not show a significant relation between ADAR1 and circRAB5A in CRC samples.

*Ns, non-significance; *, P* < 0.05; **, *P* < 0.01; ***, *P* < 0.001.
