# Supplementary material for: ADAR1-circRAB5A-BIP axis governs radiotherapy resistance in colorectal cancer through coordinating protective autophagy and apoptosis
Source: Cancer Biol Ther. 2026 Jun 21;27(1):2677975. doi: 10.1080/15384047.2026.2677975 (PMC13285610; doi:10.1080/15384047.2026.2677975)
Supplement: Supplementary material — Supplementary Figure S4.docx [file KCBT_A_2677975_SM6921.docx]

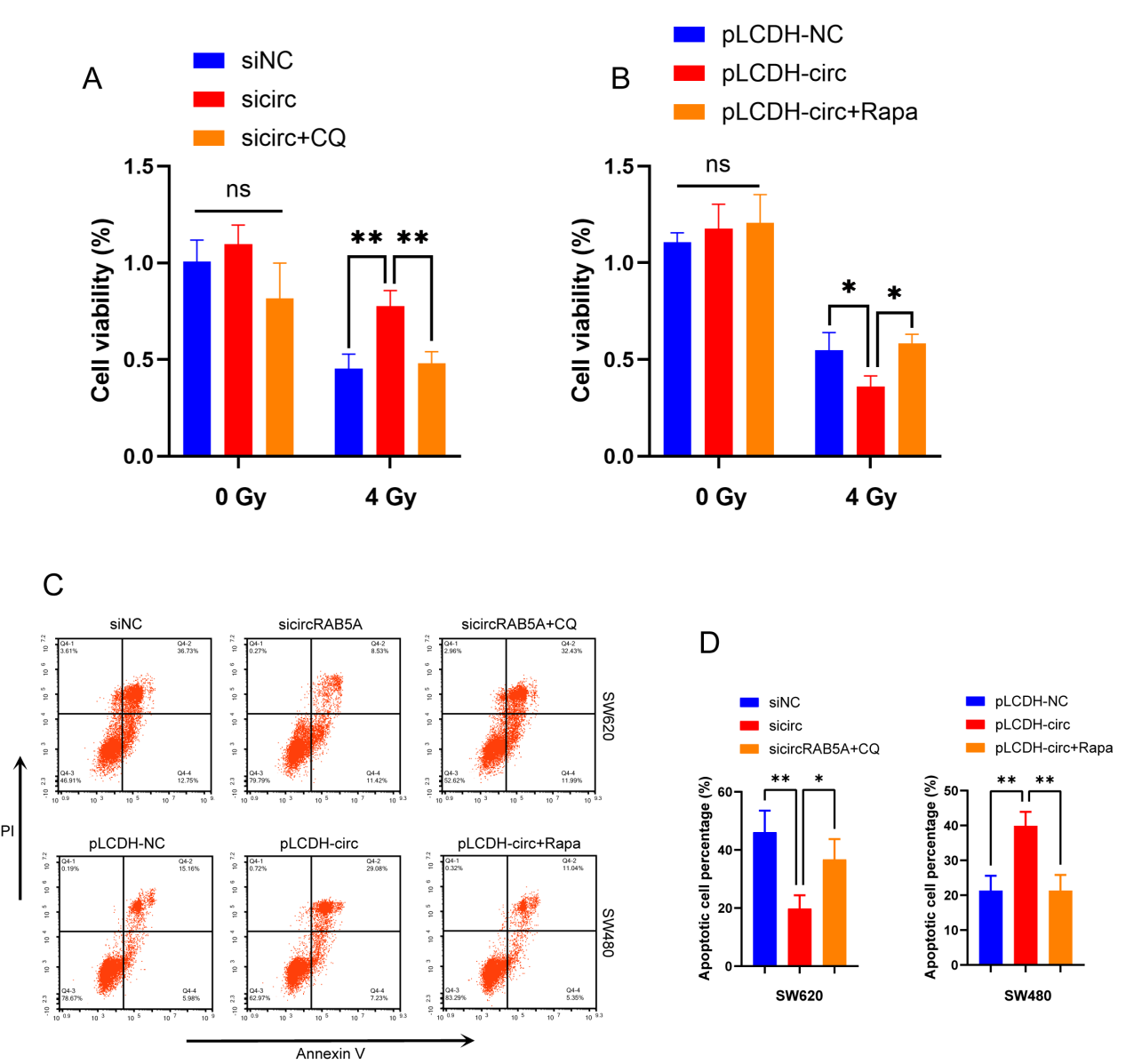


Supplementary Figure S4. CircRAB5A modulated radioresistance via regulating autophagy. We treated CRC cells with CQ or Rapamycin upon circRAB5A overexpression or knocking down. The CCK8 and apoptosis assay demonstrated that CQ abolished sicircRAB5A-induced radioresistance while Rapa counterbalanced the decreased radioresistance, induced by circRAB5A forced expression.

*Ns, non-significance; *, P* < 0.05; **, *P* < 0.01.
