# Supplementary material for: ADAR1-circRAB5A-BIP axis governs radiotherapy resistance in colorectal cancer through coordinating protective autophagy and apoptosis
Source: Cancer Biol Ther. 2026 Jun 21;27(1):2677975. doi: 10.1080/15384047.2026.2677975 (PMC13285610; doi:10.1080/15384047.2026.2677975)
Supplement: Figure Legends [file KCBT_A_2677975_SM6922.docx]

**Figure Legends**

Figure 1. CircRAB5A was downregulated in radioresistant CRC.

A: The most differentially expressed circRNA from GSE186940 is depicted as a heat map. We focused on the remarkable downregulation of circRAB5A (has-circ-0123297) was focused.

B: Validation of circRAB5A expression in 40 pairs of clinical samples using qRT-PCR. Comparison of circRAB5A levels in radiosensitive and radioresistant CRC samples.

C: qRT-PCR results showing the expression of circRAB5A in multiple CRC cell lines (HCT8, SW620, HCT116, SW480, and Caco-2) and the normal colon epithelial cell line NCM460. CircRAB5A expression was decreased in most CRC cell lines.

D: Design and verification of circRAB5A primers. Divergent primers amplified the back-splice site of circRAB5A and the sequences were verified by sequencing.

E: Amplification of circRAB5A by divergent primers in cDNA but not gDNA.

F: Result of circRAB5A FISH staining. Scale bar = 5μm.

G: Results of the sub-cellular fraction assay, GAPDH represents cytoplasmic fractions, and U6 represents the nuclear fraction.

**, P* < 0.05; **, *P* < 0.01; ***, *P* < 0.001; ****, *P* < 0.0001.

Figure 2. ADAR1 suppresses circRAB5A biogenesis via binding intron Alu Jo/Jr binding.

A: Clonogenic survival assay verified the establishment of radiation-resistant SW480 cells (SW480-IR).

B: Comparison of circRAB5A and RAB5A mRNA levels in parental SW480 and SW480-IR cells. CircRAB5A was significantly downregulated in SW480-IR cells.

C: Sketch map of full-length or Alu Jo/Jr truncated expression vectors.

D:qRT-PCR results show that only the co-existence of Alu Jo/Jr significantly promoted the biogenesis of circRAB5A.

E: qRT-PCR results of ADAR1, DHX9, and QKI expression in parental SW480 cells and SW480-IR cells. ADAR1 expression significantly increased in SW480-IR cells.

F: RIP results using the anti-ADAR1 antibody. The ADAR1 antibody significantly enriched Alu Jo/Jr sequences compared to control IgG.

G: qRT-PCR and WB results showed that overexpression of ADAR1 in SW480 cells significantly decreased circRAB5A expression.

H: qRT-PCR and WB results showed that ADAR1 knockdown increased circRAB5A expression in SW480-IR cells.

*Ns, non-significance; *, P* < 0.05; **, *P* < 0.01; ***, *P* < 0.001.

Figure 3. CircRAB5A depletion confers radioresistance of CRC cells via by regulating the autophagy-apoptosis balance.

A: Clonogenic assay results in SW620 cells showed that downregulation of circRAB5A significantly increased the radiosensitivity of SW620 cells, and cell viability increased at a treatment dose of 4 Gy.

B: Clonogenic assay results in SW480 cells showed that upregulation of circRAB5A decreased the radiosensitivity of SW480 cells, and cell viability at a treatment dose of 4 Gy was decreased.

C: WB results showed that forced expression of circRAB5A decreased LC3-II expression and promoted p62 expression. Knockdown of circRAB5A led to the opposite result.

D: Annexin V/PI double-staining results showed that after radiation treatment, the percentage of apoptotic cells significantly decreased with the reduction of circRAB5A.

E: The statistical analysis of Annexin V/PI double-staining results.

F: The statistical analysis of mCherry-GFP-LC3 dual-fluorescence reporters.

G: Forced expression of circRAB5A decreases autolysosome formation (mCherry+GFP- puncta) using mCherry-GFP-LC3 dual-fluorescence reporters.

**, P* < 0.05; **, *P* < 0.01; ***, *P* < 0.001.

Figure 4. CircRAB5A interacted with ER chaperonin, BIP in CRC cells.

A: Silver staining results of RNA pull-down assay using circRAB5A probes. Differentially enriched bands in the circ group were detected at a molecular weight of 70 kDa.

B: Verification of BIP protein enrichment in the circ group via WB detection.

C: RIP results using an anti-BIP antibody. In the BIP-bound extract, the enrichment of circRAB5A was significantly higher than that of control IgG, indicating an interaction between circRAB5A and BIP proteins in CRC cells.

D: Sketch map of wild type or truncated BIP expression vectors.

E: RNA pull-down assay using circRAB5A probes following WB of Flag antibody showed circRAB5A probe failed to pull-down Del3 truncated protein.

F: RIP results using an anti-Flag antibody. Del3 truncated protein could not precipitated circRAB5A.

**, P* < 0.05; **, *P* < 0.01.

Figure 5. CircRAB5A regulates BIP protein stability through TRIM21-mediated ubiquitination

A, C WB results of BIP protein levels after circRAB5A overexpression or knockdown. circRAB5A depletion upregulated and circRAB5A forced expression downregulated BIP protein levels.

B, D: CHX chase assay results showed that circRAB5A depletion significantly prolonged the stability of BIP protein. The statistical analysis was depicted in line chart.

E: Rescue experiments in SW480 cells showed that the degradation of BIP induced by circRAB5A overexpression was rescued after treatment with the proteasome inhibitor MG132.

F: Ubiquitination assay results showed that circRAB5A knockdown decreased, while forced expression of circRAB5A increased the ubiquitination of BIP.

G, H: Ubiquitination assay results showed that enforced TRIM21 expression in SW480 cells enhanced BIP ubiquitination, which was further increased by circRAB5A co-overexpression. In contrast, TRIM21 knockdown abrogated circRAB5A-induced BIP ubiquitination.

**, P* < 0.05; ****, *P* < 0.0001.

Figure 6. The circRAB5A/BIP axis modulates autophagy-apoptosis balance in CRC cells via the p-Akt/Beclin1 pathway

A: Results of the co-transfection experiments in CRC cells. Co-transfection of circRAB5A siRNA with BIP siRNA abolished the sicircRAB5A-induced increase in colony survival and cell viability.

B: Co-transfection with BIP reversed the circRAB5A-induced radiosensitive phenotype.

C, D: Annexin V/PI apoptosis assay results showed an increase in apoptotic cells by sicircRAB5A, and the decrease in apoptotic cells by circRAB5A forced expression was reversed by siBIP and BIP overexpression, respectively.

E, F: WB detection of LC3 and p62, and mCherry-GFP-LC3 dual-fluorescence reporters consistently indicated that the expression of BIP counterbalanced circRAB5A-mediated inhibition of autophagy.

G: WB blotting demonstrated that the circRAB5A/BIP axis conferred radioresistance in CRC cells by regulating the p-Akt/Beclin1 signaling pathway.

**, P* < 0.05; **, *P* < 0.01; ***, *P* < 0.001.

Figure 7. *In vivo* experiments revealed the role of circRAB5A/BIP axis on radioresistance.

A: *In vivo* experimental results obtained using a subcutaneous xenograft model of BALB/c nude mice. Stable knockdown of circRAB5A attenuated the effect of radiation treatment and enhanced radioresistance, as shown by changes in tumor volume and weight.

B: Effect of stable BIP knockdown on sensitivity to radiotherapy. Sh-BIP sensitized CRC cells to radiotherapy even at a low dose of radiation (2 Gy).

C, D: Immunohistochemistry (ICC) of Ki67 and TUNEL staining results showed that circRAB5A knockdown protected cell proliferation and inhibited apoptosis under radiation. However, sh-BIP yielded the opposite results.

**, P* < 0.05; **, *P* < 0.01; ***, *P* < 0.001; ****, *P* < 0.0001.
