# Supplementary material for: ADAR1-circRAB5A-BIP axis governs radiotherapy resistance in colorectal cancer through coordinating protective autophagy and apoptosis
Source: Cancer Biol Ther. 2026 Jun 21;27(1):2677975. doi: 10.1080/15384047.2026.2677975 (PMC13285610; doi:10.1080/15384047.2026.2677975)
Supplement: Supplementary material — Supplementary Tables.docx [file KCBT_A_2677975_SM6923.docx]

Supplementary Tables are available at https://doi.org/10.6084/m9.figshare.30814766.
