# Supplementary material for: ADAR1-circRAB5A-BIP axis governs radiotherapy resistance in colorectal cancer through coordinating protective autophagy and apoptosis
Source: Cancer Biol Ther. 2026 Jun 21;27(1):2677975. doi: 10.1080/15384047.2026.2677975 (PMC13285610; doi:10.1080/15384047.2026.2677975)
Supplement: Supplementary material — Supplementary Figure S6.docx [file KCBT_A_2677975_SM6924.docx]

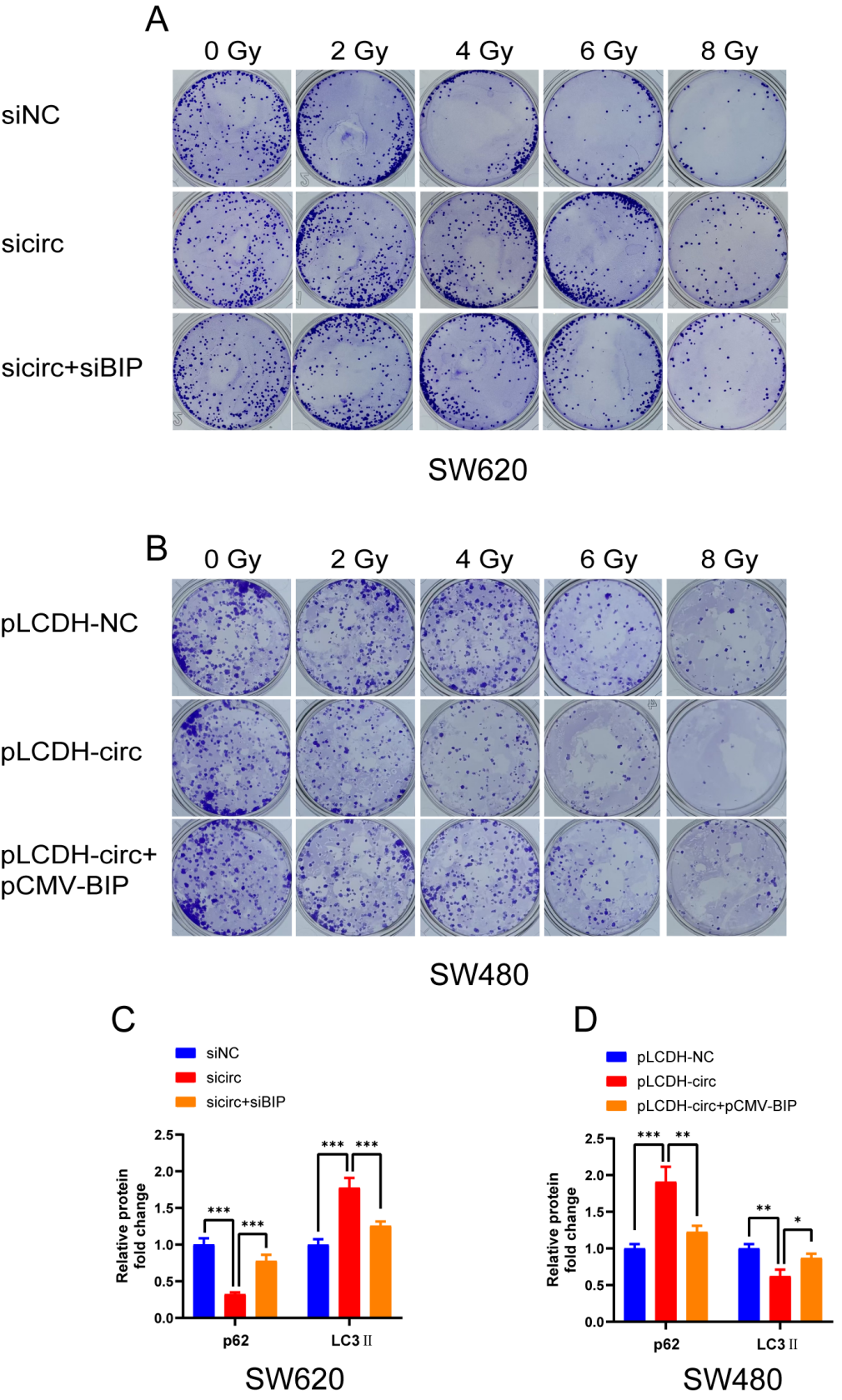


Supplementary Figure S6. The rescue experiments.

A, B: The original images of clonogenic survival assay.

C, D: The statistical analysis of WB detection in rescue experiments. SicircRAB5A increased LC3-II expression and decreased p62 levels, which were abolished by siBIP. Conversely, BIP overexpression restored circRAB5A-overexpressing induced LC3-II and p62 alterations.

**, P* < 0.05; **, *P* < 0.01; ***, *P* < 0.001.
