# Supplementary material for: ADAR1-circRAB5A-BIP axis governs radiotherapy resistance in colorectal cancer through coordinating protective autophagy and apoptosis
Source: Cancer Biol Ther. 2026 Jun 21;27(1):2677975. doi: 10.1080/15384047.2026.2677975 (PMC13285610; doi:10.1080/15384047.2026.2677975)
Supplement: Supplementary material — Supplementary Figure S7.docx [file KCBT_A_2677975_SM6925.docx]

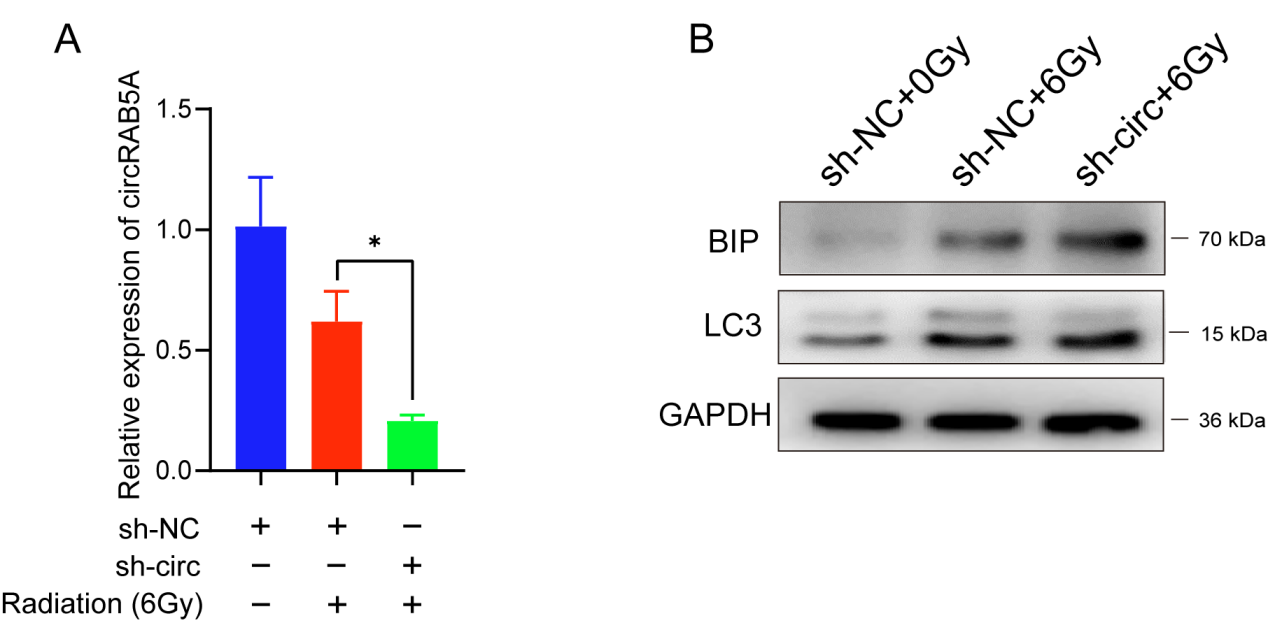


Supplementary Figure S7. qRT-PCR and Western blot results of tumor lysates from xenograft models

A. qRT-PCR result showed the stable knocking down of circRAB5A in xenografts and the expression level of circRAB5A in indicated groups.

B. WBt results showed that sh-circRAB5A increased BIP levels and LC3-II expression, and decreased p62, consistent with enhanced protective autophagy in xonografts.

**, P* < 0.05
