# Supplementary material for: ADAR1-circRAB5A-BIP axis governs radiotherapy resistance in colorectal cancer through coordinating protective autophagy and apoptosis
Source: Cancer Biol Ther. 2026 Jun 21;27(1):2677975. doi: 10.1080/15384047.2026.2677975 (PMC13285610; doi:10.1080/15384047.2026.2677975)
Supplement: Supplementary Figure S5.docx [file KCBT_A_2677975_SM6926.docx]

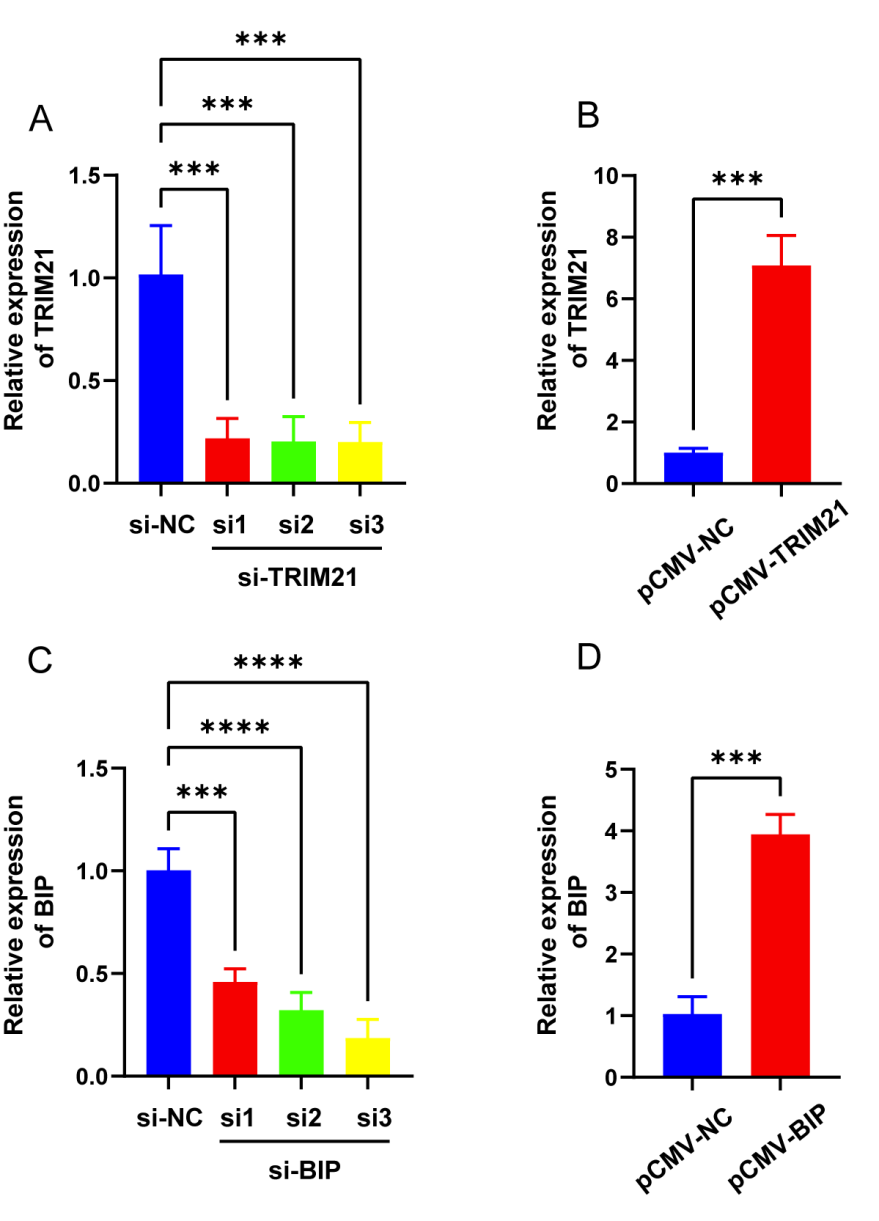


Supplementary Figure S5. Validation of TRIM21 and BIP knockdown and overexpression efficiency.

A. qRT-PCR results showed that siTRIM21 efficiently knocked down TRIM21 mRNA level.

B. qRT-PCR results showed pCMV-TRIM21 significantly induced the forced expression of TRIM21.

C. qRT-PCR results showed that siBIP efficiently knocked down BIP mRNA level.

D. qRT-PCR results showed pCMV-BIP significantly induced the forced expression of BIP.

***, *P* < 0.001; ****, *P* < 0.0001;
